# Different MRI-based radiomics machine learning models to predict CD3+ tumor-infiltrating lymphocytes in rectal cancer

**DOI:** 10.3389/fonc.2025.1509207

**Published:** 2025-04-28

**Authors:** Weili Ma, Chuanling Hou, Minxia Yang, Yuguo Wei, Jiwei Mao, Le Guan, Zhenhua Zhao

**Affiliations:** ^1^ Department of Radiology, Shaoxing People’s Hospital, Key Laboratory of Functional Molecular Imaging of Tumor and Interventional Diagnosis and Treatment of Shaoxing City, Shaoxing, China; ^2^ Department of Pathology, Shaoxing People’s Hospital, Shaoxing, China; ^3^ Advanced Analytics, Global Medical Service, GE Healthcare, Hangzhou, China; ^4^ Department of Radiotherapy, Shaoxing People’s Hospital, Shaoxing, China

**Keywords:** CD3, tumor-infiltrating lymphocytes, radiomics, rectal cancer, machine learning

## Abstract

**Objectives:**

This study aimed to develop and evaluate multiple machine learning models utilizing contrast-enhanced T1-weighted imaging (T1-CE) to differentiate between low-/high-infiltration of total T lymphocytes (CD3) in patients with rectal cancer.

**Methods:**

We retrospectively selected 157 patients (103 men, 54 women) with pathologically confirmed rectal cancer diagnosed between March 2015 and October 2019. The cohort was randomly divided into a training dataset (n=109) and a test dataset (n=48) for subsequent analysis. Seven radiomic features were selected to generate three models: logistic regression (LR), random forest (RF), and support vector machine (SVM). The diagnostic performance of the three models was compared using the DeLong test. Additionally, Kaplan–Meier analysis was employed to assess disease-free survival (DFS) in patients with high and low CD3+ tumor-infiltrating lymphocyte (TIL) density.

**Results:**

The three radiomics models performed well in predicting the infiltration of CD3+ TILS, with area under the curve (AUC) values of 0.871, 0.982, and 0.913, respectively, in the training set for the LR, RF, and SVM models. In the validation set, the corresponding AUC values were 0.869, 0.794, and 0.837, respectively. Among the radiomics models, the LR model exhibited superior diagnostic performance and robustness. The merged model, which integrated radiomics features from the SVM model and clinical features from the clinical model, outperformed the individual radiomics models, with AUCs of 0.8932 and 0.8829 in the training and test cohorts, respectively. Additionally, a lower expression level of CD3+ TILs in the cohort was independently correlated with DFS (*P* = 0.0041).

**Conclusion:**

The combined model demonstrated a better discriminatory ability in assessing the abundance of CD3+ TILs in rectal cancer. Furthermore, the expression of CD3+ TILs was significantly correlated with DFS, highlighting its potential prognostic value.

**Advances in knowledge:**

This study is the first attempt to compare the predictive TILs performance of three machine learning models, LR, RF, and SVM, based on the combination of radiomics and immunohistochemistry. The MRI-based combined model, composed of radiomics features from the SVM model and clinical features from the clinical model, exhibited better discriminatory capability for the expression of CD3+ TILs in rectal cancer.

## Introduction

Colorectal cancer remains one of the most common causes of cancer-related mortality worldwide (1, 2). The prognosis of the disease is largely determined by the stage at which it is diagnosed (3). Early detection and appropriate treatment are critical in improving patient outcomes. In recent years, the increasing adoption of immunotherapy, particularly immune checkpoint inhibitors (ICIs), has shown promise in clinical practice, with several landmark trials demonstrating significant therapeutic efficacy, This has opened the possibility of innovative treatment strategies that can modulate immune responses to better target cancer cells (4, 5). Among these, tumor-infiltrating lymphocytes (TILs) have emerged as a potential predictive biomarker for treatment response, including neoadjuvant therapy in locally advanced rectal cancer (6–9).

High levels of TILs, which are located in the tumor microenvironment, have been associated with improved immune responses and better treatment outcomes in various cancers (10). For example, studies have demonstrated that higher levels of TILs correlate with better responses to neoadjuvant chemotherapy in breast cancer, particularly in HER2-positive cases (5, 11). However, despite advances in treatment, up to 30% of patients with rectal cancer still experience poor prognosis, including distant metastasis or local recurrence, often occurring within a few years of treatment. These findings highlight the need for more effective patient assessment methods to identify individuals who are most likely to benefit from immunotherapy and to optimize treatment outcomes.

Recent research has increasingly focused on the relationship between radiomics and tumor TIL levels, especially CD8, with studies exploring this association in breast cancer (12), rectal cancer after chemoradiation (13), and pancreatic cancer (14). In particular, Huang et al. demonstrated that texture features extracted from dynamic contrast-enhanced magnetic resonance imaging (DCE-MRI) are correlated with CD8+ and CD4+ T lymphocytes, providing insights into the immune microenvironment’s histopathological features in advanced gastric cancer (15). Despite the progress in immunotherapy, the potential correlation between imaging data and the immune microenvironment in cancer remains an area that is only partially understood (16).

Given the importance of TILs in predicting treatment response and prognosis, we hypothesized that radiomics could be used to predict the abundance of CD3+ TILs in rectal cancer. The primary objective of our research was to develop accurate risk stratification models to differentiate between a low- and high-abundance of CD3+ TILs in rectal cancer, which could ultimately aid in better treatment decision-making and personalized therapeutic strategies.

## Materials and methods

### Patient selection

Our research has been approved by the ethics committee. The data of patients diagnosed with rectal cancer, who underwent surgical resection between March 2015 to October 2019, were retrospectively collected for the construction of the radiomics model. We included patients who met the following criteria: (a) underwent surgical resection and (b) preoperative T1-weighted imaging (T1-CE) performed within 3 weeks. Exclusion criteria were (a) incomplete clinical data; (b) incomplete pathology report; and (c) tumor-related treatment before MRI examination. The final cohort consisted of 157 patients, who were randomly divided into a training set of 109 patients and a test set of 48 patients.

### MRI examination

The rectal MRI imaging was performed using a 3T unit (Verio, Siemens, Germany) equipped with a 12-channel body coil. The examinations included T1-CE, high-resolution axial T2-weighted imaging, and diffusion-weighted imaging (DWI). Detailed information on the standardized imaging protocols is provided in **Supplementary Material**.

### Pathology

**Figure 1:**
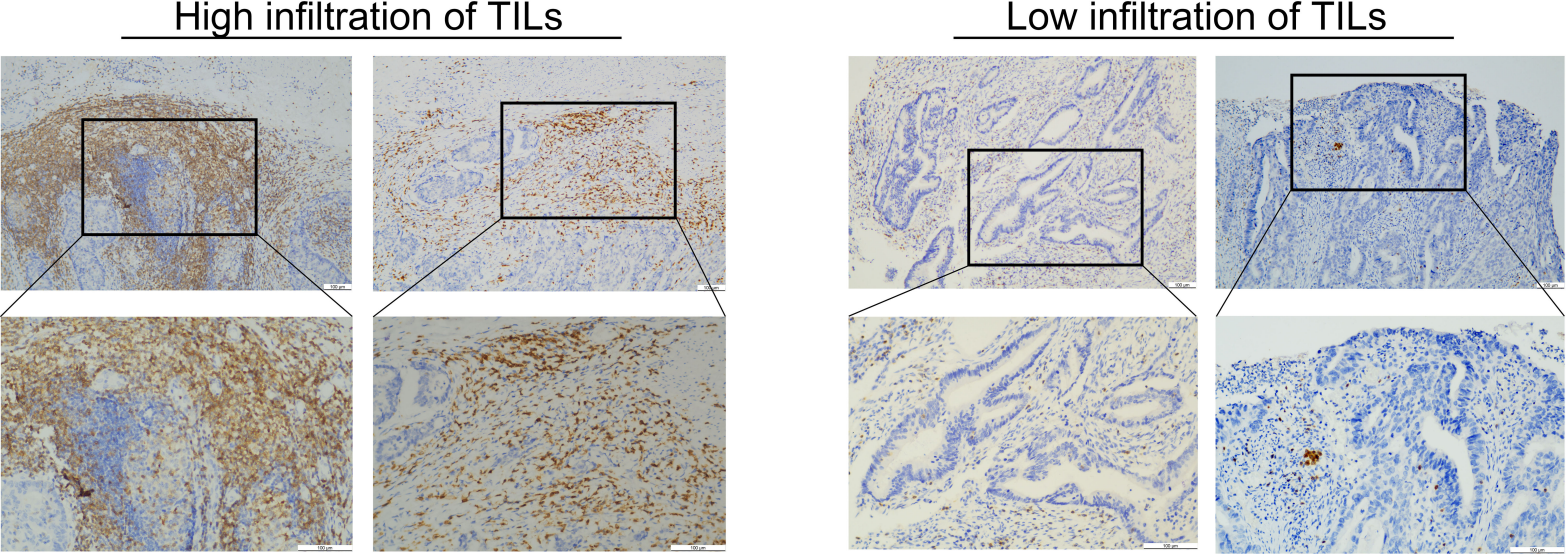
This image shows the evaluation of the TILs for rectal cancer. The left image shows high TIL infiltration. The right image shows low TIL infiltration.

All wax block samples were sectioned into 2 μm thick slices by professional technicians. The slices were then placed in an oven at 56°C overnight. CD3 antibody (ZM-0417, Zhongshan Jinqiao, Beijing, China) was reserved at 1:200. Bond ™ Polymer Refine Detection immunoassay kit (DS9800, Leica Biosystems, United Kingdom). The entire immunohistochemistry (IHC) process was carried out using the BOND-MAX Fully Automated IHC and ISH Staining System. Finally, all sections were dehydrated, coverslipped, and embedded in neutral resin. Two senior pathologists independently reviewed all IHC slides and randomly selected five High Power Field (HPFs) to evaluate the expression of CD3+ TILs, based on the percentage of positive lymphocytes in the stroma. Patients were stratified into high or low CD3+ TIL expression groups using the lower quartile as the threshold. The assessment of TILs in rectal cancer is shown in **Figure 1**.

### Tumor segmentation and feature extraction

**Figure 2:**
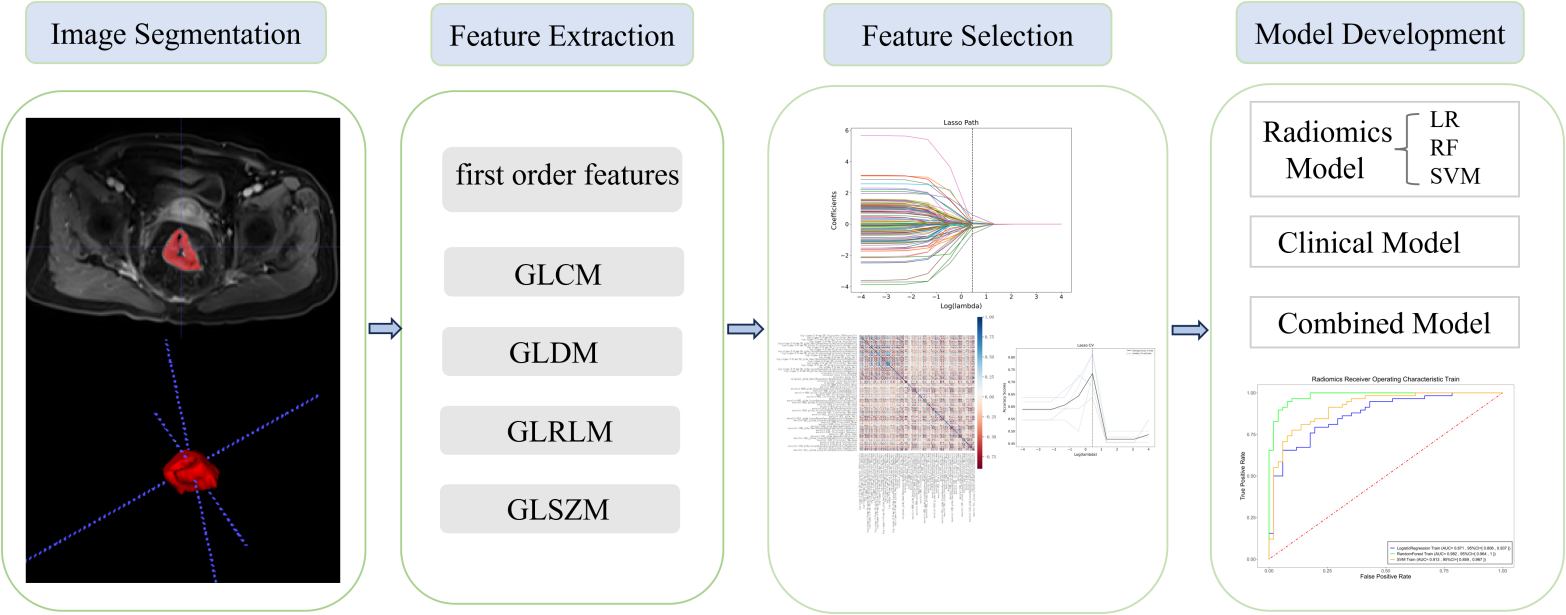
Outline of interest and workflow of the present study.

The regions of interest (ROIs) of 157 lesions were segmented using ITK-SNAP (www.itksnap.org), an open-source software, on each CE-T1WI slice. Two radiologists, each with 3 years of experience in MRI, manually delineated the entire area within the rectal wall, covering the entire tumor and excluding necrotic tissue and bleeding (**Figure 2**). Clinical and histopathological data were collected by radiologists in a blinded manner, with the exception of information regarding the diagnosis of rectal cancer.

All radiomics features were extracted using the Pyradiomics package (http://www.PyRadiomics.readthedocs.io/en/latest/) in Python (3.8.0). A total of 1,132 radiomics features, including 234 first-order features, 286 gray level co-occurrence matrix (GLCM) features, 182 gray level dependence matrix (GLDM) features, 208 gray level run length matrix (GLRLM) features, 208 gray level size zone matrix (GLSZM) features, and 14 shape features were extracted from the original images. Before feature extraction, the MRI images were standardized using the z-score normalization method to ensure a consistent distribution of image intensities.

### Radiomic feature selection and radiomics signature building

Only features with high interobserver reproducibility (intraclass correlation coefficient >0.75) were retained for subsequent analysis. Radiomics feature clusters with a correlation coefficient less than 0.9 (Spearman’s r<0.9) were kept to eliminate redundancy between features.

The least absolute shrinkage and selection operator (LASSO) method with fivefold cross-validation was then employed to identify the most predictive features from the training set. A radiomics score for each patient was calculated based on the linear combination of the selected imaging features.

Three radiomics models [logistic regression (LR), random forest (RF), and support vector machine (SVM)] and one clinical model were developed for predicting CD3+ TILs. Consequently, four integrated models were generated by combining the clinical model and the radiomics models.

### Statistical analyses

Statistical analyses were performed using R (version 3.5.3; http://www.r-project.org). Datasets with a normal distribution were summarized using the mean and standard deviation, while categorical variables were presented using medians and ranges. Spearman’s correlation was used to assess the degree of correlation between features. Model discrimination was evaluated using the area under the curve (AUC). Additionally, sensitivity, specificity, positive predictive value (PPV), and negative predictive value (NPV) were calculated. The prediction performance of four radiomics models was assessed and compared using the DeLong method (17, 18). The improvement in the integrated models was evaluated by assessing the integrated differentiation improvement (19). Kaplan–Meier survival analysis was performed to evaluate disease -free survival (DFS) probabilities and differences between the high and low CD3 expression groups were compared using the log-rank test.

## Results

### Patient characteristics

The patients were stratified into a high-density group and a low-density group based on the lower quartile of CD3 expression (**Figure 1**). Of the 157 patients, 73 were classified into the low-expression group and 84 into the high-expression group. No statistically significance differences were observed between the training group and the validation groups, except for gender (*P*=0.045). The characteristics of the included for analysis patients are detailed in **Table 1**.

**Table 1:** Clinical characteristics in the training and test cohorts.

Characteristic	Training Set (n=109)	Test Set (n=48)	*P* Value
Sex
Man	77	26	0.045
Female	32	22	
Age	66.6±10.5	64.9±10.5	0.833
CEA(>10ng/ml)
Negative	98	40	0.245
Positive	11	8	
CA199(>60 ng/ml)
Negative	101	44	0.829
Positive	8	4	
N
Negative	35	23	0.059
Positive	74	25	
Differentiation
Low	5	1	0.763
High/Middle	104	47	
EVI
Negative	102	38	0.007
Positive	7	10	
Tumor size (cm)†	3.8±1.5	3.9±1.4	0.959
T stage
I–II	35	14	0.714
III–IV	74	34	

EVI, extramural venous invasion.

### Feature selection

**Figure 3:**
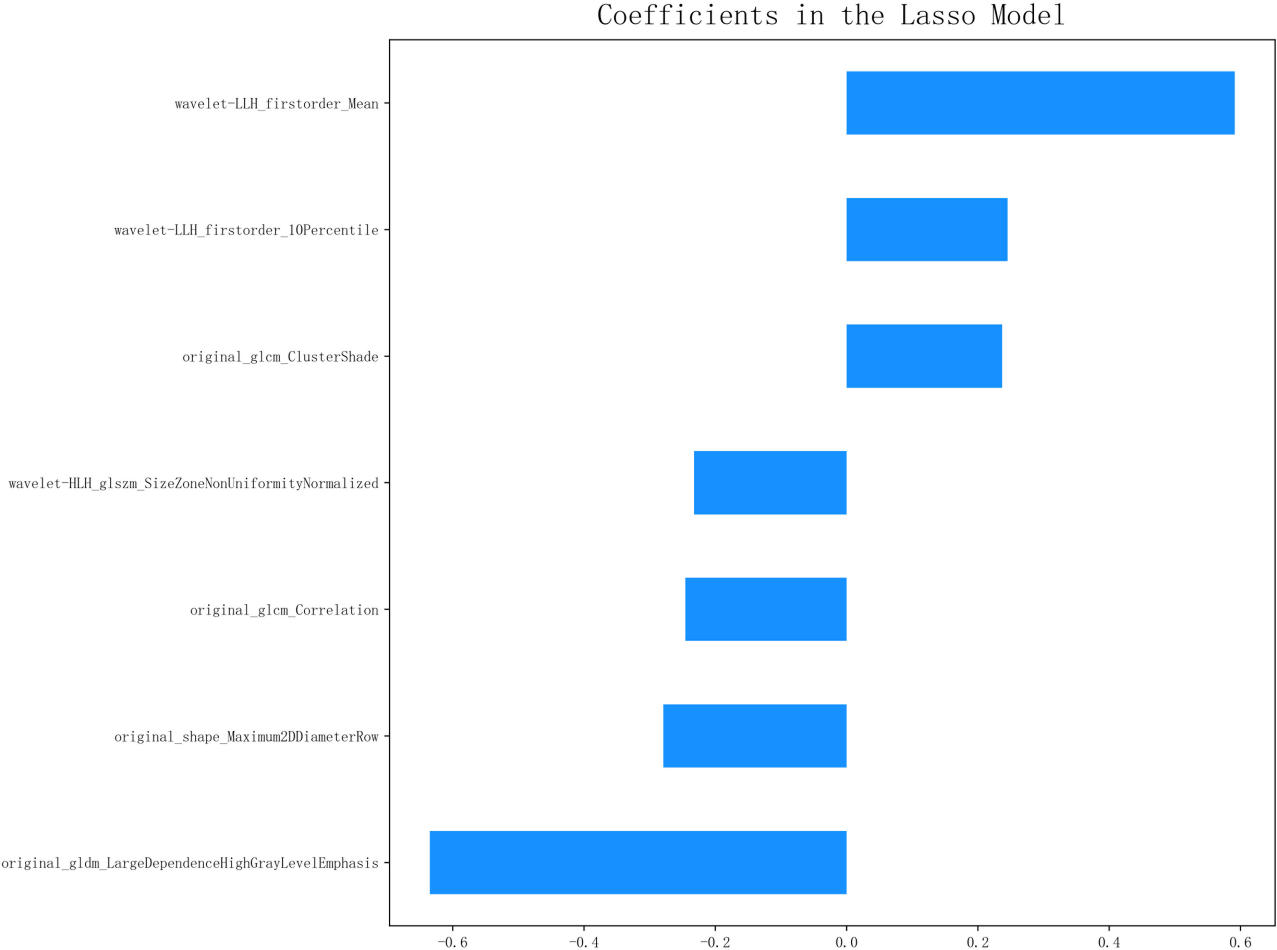
The selected radiomics features and their corresponding coefficients.

A total of 1,132 radiomics features were extracted from the ROIs following tumor segmentation (**Figure 2**). To ensure high stability and reproducibility, 292 features with an ICC >0.75 were retained from all radiomics features. Furthermore, 107 features exhibiting low correlations with the voxel value of each lesion were subjected to LASSO models to select the optimal features. Ultimately, seven radiomics features were selected to construct radiomics prediction models. Selected radiomics features and their corresponding coefficients are described in **Figure 3**. Among the clinical features, only the N stage feature was retained. The combined model was developed using the seven radiomics features and one clinical feature. The retained features of the screening process are shown in the **Supplementary Material**.

### Performance of the clinical and radiomics models

**Figure 4:**
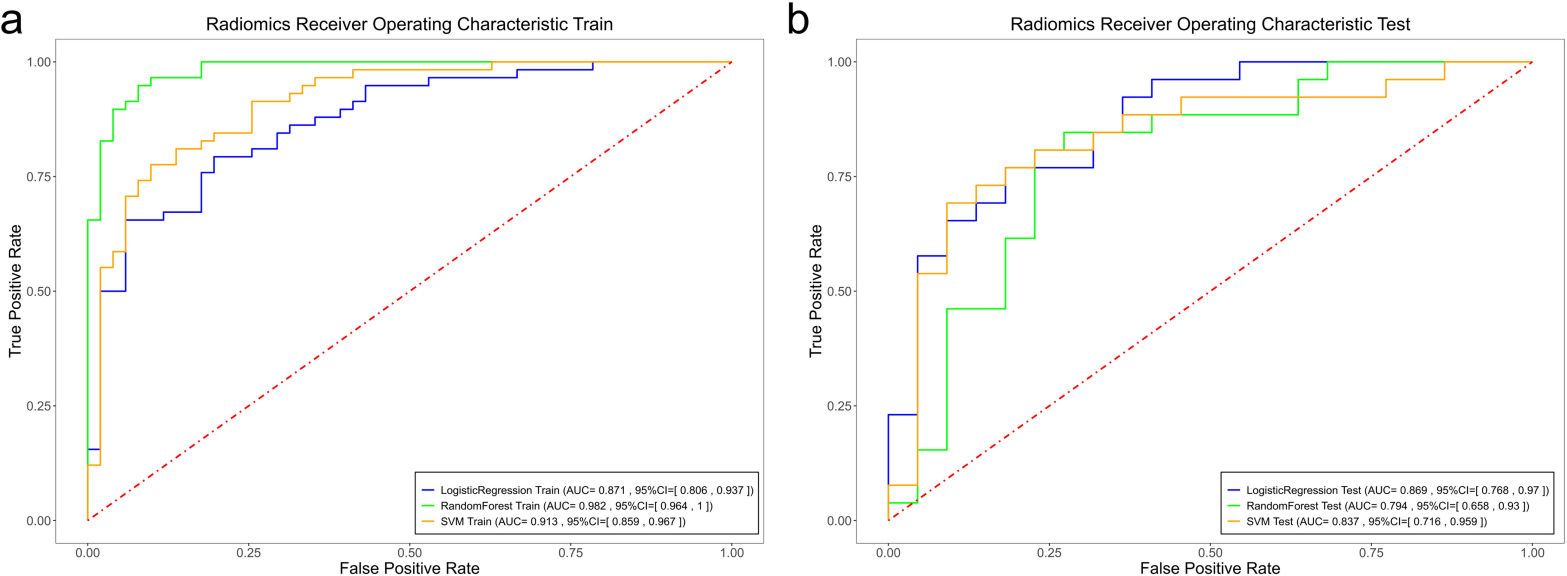
Comparison of the ROC curves of the support vector machine (SVM), random forest (RF), and logistic regression (LR) models in the training set **(a)** and the validation set **(b)**.

**Figure 5:**
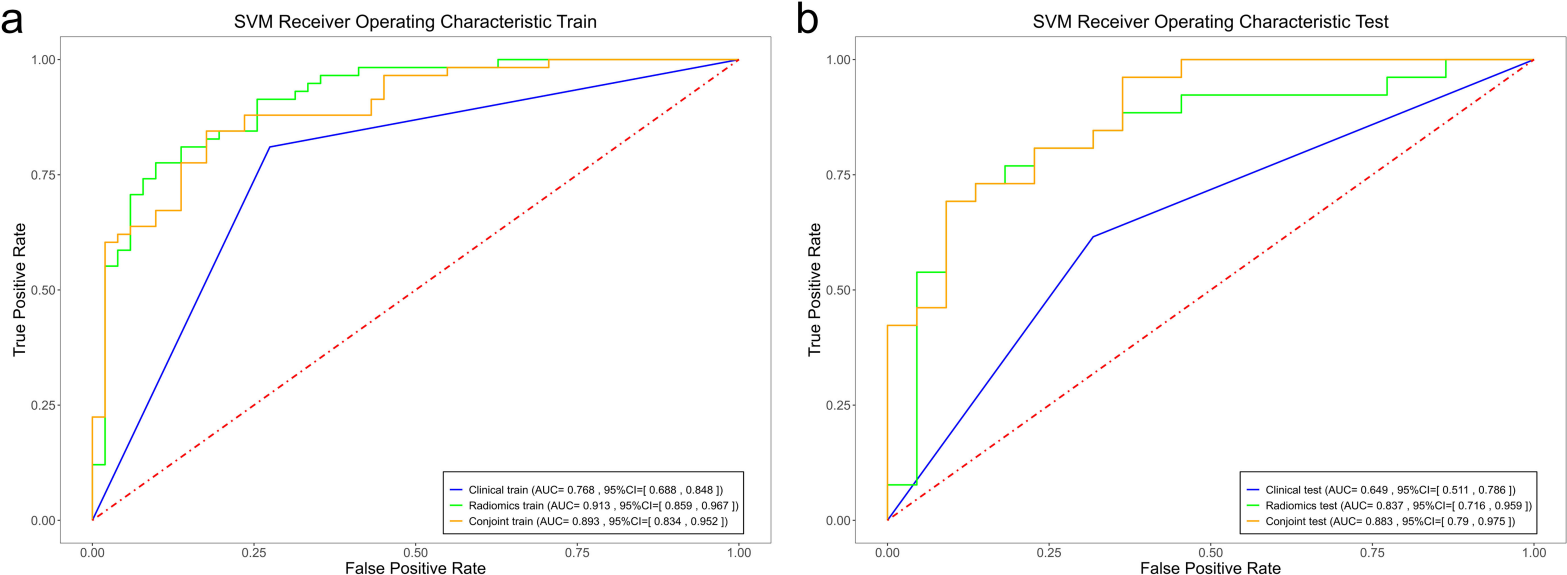
Comparison of the ROC curves of the support vector machine (SVM), random forest (RF), and logistic regression (LR) models in the training set **(a)** and the validation set **(b)**.

Among the radiomics models, the RF radiomics model achieved the highest AUC value of 0.982 [95% confidence interval (CI): 0.964, 1] in the training set, and the LR radiomics model achieved the highest AUC value of 0.869 (95% CI:0.768, 0.97) in the validation set (**Figure 4**). **Table 2** shows the detailed prediction performance of various radiomics models. For the clinical model, the AUC, sensitivity, and specificity in the training set were 0.7679 (95% CI: 0.688, 0.848), 0.8103 and 0.7255, respectively. In the test set, these values were 0.6486 (95% CI:0.511, 0.786), 0.6154, and 0.6818, respectively. The combined model, which integrated the SVM model and clinical model, achieved the highest discriminatory ability (AUC, training cohort: 0.8932; test cohort: 0.8829) and robustness for expression of CD3+ TILs in rectal cancer (**Figure 5**).

**Table 2:** Performance models for predicting CD3+ TILs in rectal cancer.

odel		AUC	SEN	SPE	PPV	NPV
LR	train	0.8712	0.7931	0.8039	0.8214	0.7736
	test	0.8689	0.8462	0.6818	0.7586	0.7895
RF	train	0.9824	0.9483	0.9216	0.9322	0.94
	test	0.7937	0.7692	0.7727	0.8	0.7391
SVM	train	0.9131	0.7759	0.902	0.9	0.7797
	test	0.8374	0.8077	0.7273	0.7778	0.7619
Clinical	train	0.7679	0.8103	0.7255	0.7705	0.7708
	test	0.6486	0.6154	0.6818	0.6957	0.6
LR+ clinical	train	0.8999	0.8103	0.8627	0.8704	0.8
	test	0.8724	0.7308	0.8182	0.8261	0.72
RF+ clinical	train	0.9337	0.7586	0.9608	0.9565	0.7778
	test	0.8575	0.7308	0.8636	0.8636	0.7308
SVM+ clinical	train	0.8932	0.8448	0.8235	0.8448	0.8235
	test	0.8829	0.7692	0.7727	0.8	0.7391

LR, logistic regression; RF, random forest; SVM, support vector machine; AUC, area under the curve; PPV, positive prediction value; NPV, negative prediction value.

### Performance comparison

However, no significant differences (*P >*0.05) in AUC were observed among the four radiomics models. For the f LR and clinical combination model, both the training and validation groups showed a slight improvement in AUC (0.0287, 0.0035) (**Table 2**). In the combinations of SVM and RF with the clinical model, the validation group demonstrated an improvement in AUC (0.9824 vs. 0.9337 and 0.9131 vs. 0.8932), whereas the training group did not show similar improvements (0.8557 vs. 0.7937 and 0.8374 vs. 0.8829).

### Predictive value of CD3 for individual DFS

**Figure 6:**
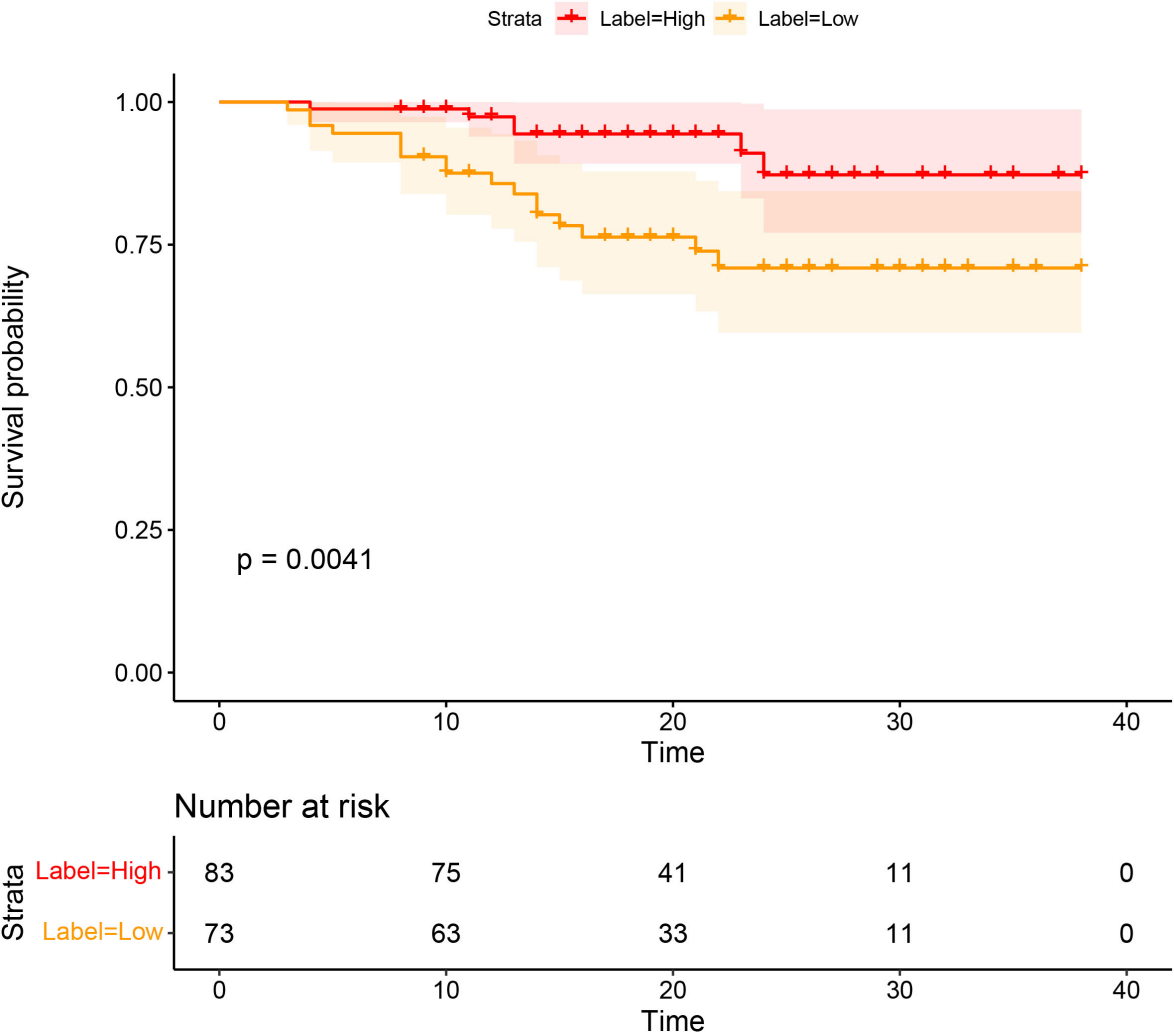
Survival analyses based on rectal cancer disease-free survival (DFS) according to CD3 infiltration.

Significant differences in DFS were observed between the high and low CD3 expression groups (P=0.0041). The Kaplan–Meier survival analysis of DFS according to CD3 expression is shown in **Figure 6**.

## Discussion

In this primary study, we developed and validated radiomics prediction models and integrated models that combined clinical factors with radiomics features to predict CD3+ TILs in rectal cancer using preoperative T1-CE. Although several efforts have been made in various cancers to associate radiomics features with immunohistochemical features to predict TIL levels, to the best of our knowledge, this study is the first to integrate both immunohistochemical and radiomics features for TIL prediction in rectal cancer. This primary research demonstrates that radiomics models, especially the LR model, exhibited the highest predictive performance. However, no significant statistical differences were observed between the three radiomics model. Notably, the combined model composed of SVM and the clinical model showed the highest discriminative capability (AUC, training cohort: 0.8932; test cohort: 0.8829) and robustness for the expression of CD3+ TILs in rectal cancer. In line with our results, Zheng et al. (20) reported that a combined model using SVM and a clinical model exhibited the highest differentiating value (with AUC 0.904 in the training cohort and 0.854 in the validation cohort) compared to the clinical and other radiomics models. Additionally, only the LR model combined with the clinical model improved the AUC value in both the training and validation groups. This analysis suggests that the clinical feature was of limited value in improving the model.

In recent years, immunotherapy and immune checkpoint blockade (ICB) for the treatment of breast cancer patients have raised concerns in clinical practice (21). Tumor-infiltrating immune cells play a crucial role in this response, and TILs make up the majority of these immune cells (22). It is now known that the success of immunotherapy requires pre-existing anti-tumor immunity, which can reflect an individual’s immune tumor response and has strong prognostic and predictive significance. The number of TILs may be a significant predictor of the response to cytotoxic treatments such as chemotherapy and radiotherapy and is assumed to be associated with the mechanisms regulating cancer growth, progression, and metastasis (23, 24). Lujiao et al. (25) used computed tomography (CT) to predict non-small cell lung cancer CD3+ and CD8+ TIL levels and developed a classifier with AUCs of 0.94 and 0.87 in the validation sets, respectively. Huang et al. (15) discovered that texture features extracted from DCE-MRI are correlated with CD8+ and CD4+ T lymphocytes in advanced gastric cancer, with diagnostic efficiencies of 0.863 and 0.856, respectively. The above results are generally consistent with our findings. Yun et al. (26) reported that an XGBoost-based radiomics model can effectively predict TILs in pancreatic ductal adenocarcinoma, with AUCs of 0.93 and 0.79 in the training and validation sets, respectively. Regarding the prediction performance of CD3, the three models showed efficacy rates of 0.869, 0.794, and 0.837, respectively, in the validation group.

Another key focus of our investigation was the role of stromal TILs, with a particular emphasis on their spatial distribution at the invasive margin. TILs in the stromal region play a critical role in tumor growth, progression, invasion, and metastasis (27, 28). Importantly, the density of intratumoral TILs is generally much lower than that of stromal TILs, making stromal TILs a more reliable biomarker for immunotherapy prediction (5). Recent studies have also classified cancers as “hot” tumors (rich infiltration of T lymphocytes) and “cold” tumors (poor infiltration of T lymphocytes) (29), which can help predict survival and treatment response (26, 30). Our findings support this discovery, as we observed that DFS was significantly associated with the expression of CD3+ TILs. Specifically, the high CD3 expression group had significantly better DFS than the low CD3 expression group. Previous studies have reported that the radiomics data from various tumor types may predict TIL density and correlate with patients’ responses to immunotherapy (31, 32). Changhee et al. (33) found that patients predicted to have a lower expression of TILs (median 4.0 months vs. 2.1 months, P=0.002) had significantly longer progression-free survival compared to those with higher predicted TIL expression (≥ median). Compared to patients with progressive disease, those who experienced an ICI response or stable disease had higher predicted TIL expression, which was the best response (P=0.001 and P=0.036, respectively). Another study (34) found that a predictive model that combines pre-treatment MRI radiological features with TIL levels can improve the accuracy of predicting pCR to NAST in patients with TNBC (P<. 001, 90.9% PPV, 81.4% NPV, and AUC 0.752). These studies found that tumor homogeneity was associated with high TIL infiltration, supporting the potential for radiomics to guide immunotherapy stratification and identify patients who may benefit from such treatments (35, 36).

Our study had several limitations. First, it was a retrospective and single-center study with a relatively small sample size. To improve the robustness of the model, further studies with a large sample size from multiple institutions are needed to ensure better robustness of the model. Second, there may be some controversy regarding the limits of manual delineation. Third, our conclusion that the radiomics model can predict the immune cells was based on circumstantial evidence, so additional validation and exploration are needed to confirm these findings.

## Conclusion

In summary, a combined model that integrated SVM and a clinical feature exhibited better discriminative capability for the expression of CD3+ TILs in rectal cancer. This predictive model has the potential to provide an approach to precision medicine and may assist in the selection of candidates for immunotherapy.

## Data Availability

The original contributions presented in the study are included in the article/**Supplementary Material**. Further inquiries can be directed to the corresponding author.
