# Supplementary material for: Different MRI-based radiomics machine learning models to predict CD3+ tumor-infiltrating lymphocytes in rectal cancer
Source: Front Oncol. 2025 Apr 28;15:1509207. doi: 10.3389/fonc.2025.1509207 (PMC12066337; doi:10.3389/fonc.2025.1509207)
Supplement: Supplementary file 1 [file DataSheet1.docx]

***Supplementary Material 1***

All imaging data were obtained using a 3.0-T MRI scanner (Verio, Siemens, Germany) with a 12-channel phased-array body coil. Patients were required to fast for at least 8 h to empty the gastrointestinal tract and inject antispasmodic medication (Anisodamine, Minsheng, Hangzhou, China) before the MRI examination to reduce gastrointestinal artifacts. During the MRI scan, the patient was placed in the supine position, and the positioning line was located on the xiphoid process. All patients underwent a routine plain scan (T1WI, T2WI with fat suppression, T2WI, ADC, DWI) before the CE-T1WI scan and a multi-angle cross-sectional T1WI in the axial plane scan (repetition time/echo time, 3.48 ms/1.3 ms; layer thickness, 3 mm; field of view, 260 mm × 260 mm; matrix, 202 × 288). CE-T1WI, the contrast agent was meglumine gadolinate, the total amount was 0.1 mmol/kg. After 40 seconds of injection, image acquisition was performed, the parameters were TR 3.56 ms, TE 1.39 ms, FOV 34.9 cm × 34.9 cm, NSA=1, layer thickness 4.5 mm, no layer spacing.

Table E1 (online)

| character | coe |
| --- | --- |
| original_glcm_ClusterShade | 0.236801 |
| original_glcm_Correlation | -0.24556 |
| original_gldm_LargeDependenceHighGrayLevelEmphasis | -0.63509 |
| original_shape_Maximum2DDiameterRow | -0.27925 |
| wavelet-HLH_glszm_SizeZoneNonUniformityNormalized | -0.2324 |
| wavelet-LLH_firstorder_10Percentile | 0.244896 |
| wavelet-LLH_firstorder_Mean | 0.591032 |

Table E2 (online)

| ***first selected*** | |  |  |  |  |  |
| --- | --- | --- | --- | --- | --- | --- |
| 0 | log-sigma-2-0-mm-3D_firstorder_10Percentile | | | | |  |
| 1 | log-sigma-2-0-mm-3D_firstorder_90Percentile | | | | |  |
| 2 | log-sigma-2-0-mm-3D_firstorder_Energy | | | |  |  |
| 3 | log-sigma-2-0-mm-3D_firstorder_Entropy | | | |  |  |
| 4 | log-sigma-2-0-mm-3D_firstorder_Kurtosis | | | |  |  |
| 5 | log-sigma-2-0-mm-3D_firstorder_Maximum | | | | |  |
| 6 | log-sigma-2-0-mm-3D_firstorder_Mean | | | |  |  |
| 7 | log-sigma-2-0-mm-3D_firstorder_Minimum | | | | |  |
| 8 | log-sigma-2-0-mm-3D_firstorder_Range | | | |  |  |
| 9 | log-sigma-2-0-mm-3D_firstorder_Skewness | | | | |  |
| 10 | log-sigma-2-0-mm-3D_glcm_ClusterProminence | | | | |  |
| 11 | log-sigma-2-0-mm-3D_glcm_ClusterShade | | | | |  |
| 12 | log-sigma-2-0-mm-3D_glcm_Correlation | | | |  |  |
| 13 | log-sigma-2-0-mm-3D_glcm_Idmn | | | |  |  |
| 14 | log-sigma-2-0-mm-3D_glcm_Imc1 | | | |  |  |
| 15 | log-sigma-2-0-mm-3D_glcm_JointEnergy | | | |  |  |
| 16 | log-sigma-2-0-mm-3D_gldm_DependenceEntropy | | | | |  |
| 17 | log-sigma-2-0-mm-3D_gldm_DependenceNonUniformityNormalized | | | | | |
| 18 | log-sigma-2-0-mm-3D_gldm_LargeDependenceHighGrayLevelEmphasis | | | | | |
| 19 | log-sigma-2-0-mm-3D_gldm_LargeDependenceLowGrayLevelEmphasis | | | | | |
| 20 | log-sigma-2-0-mm-3D_gldm_SmallDependenceLowGrayLevelEmphasis | | | | | |
| 21 | log-sigma-2-0-mm-3D_glszm_LargeAreaEmphasis | | | | |  |
| 22 | log-sigma-2-0-mm-3D_glszm_LargeAreaHighGrayLevelEmphasis | | | | | |
| 23 | log-sigma-2-0-mm-3D_glszm_LargeAreaLowGrayLevelEmphasis | | | | | |
| 24 | log-sigma-2-0-mm-3D_glszm_SizeZoneNonUniformity | | | | | |
| 25 | log-sigma-2-0-mm-3D_glszm_SizeZoneNonUniformityNormalized | | | | | |
| 26 | log-sigma-3-0-mm-3D_firstorder_10Percentile | | | | |  |
| 27 | log-sigma-3-0-mm-3D_firstorder_90Percentile | | | | |  |
| 28 | log-sigma-3-0-mm-3D_firstorder_Kurtosis | | | |  |  |
| 29 | log-sigma-3-0-mm-3D_firstorder_Maximum | | | | |  |
| 30 | log-sigma-3-0-mm-3D_firstorder_Minimum | | | | |  |
| 31 | log-sigma-3-0-mm-3D_firstorder_Range | | | |  |  |
| 32 | log-sigma-3-0-mm-3D_firstorder_RobustMeanAbsoluteDeviation | | | | | |
| 33 | log-sigma-3-0-mm-3D_firstorder_Skewness | | | | |  |
| 34 | log-sigma-3-0-mm-3D_glcm_ClusterShade | | | | |  |
| 35 | log-sigma-3-0-mm-3D_glcm_Correlation | | | |  |  |
| 36 | log-sigma-3-0-mm-3D_glcm_Idmn | | | |  |  |
| 37 | log-sigma-3-0-mm-3D_glcm_Imc2 | | | |  |  |
| 38 | log-sigma-3-0-mm-3D_glcm_InverseVariance | | | | |  |
| 39 | log-sigma-3-0-mm-3D_gldm_LargeDependenceHighGrayLevelEmphasis | | | | | |
| 40 | log-sigma-3-0-mm-3D_gldm_LargeDependenceLowGrayLevelEmphasis | | | | | |
| 41 | log-sigma-3-0-mm-3D_gldm_SmallDependenceLowGrayLevelEmphasis | | | | | |
| 42 | log-sigma-3-0-mm-3D_glrlm_RunEntropy | | | |  |  |
| 43 | log-sigma-3-0-mm-3D_glszm_GrayLevelVariance | | | | |  |
| 44 | log-sigma-3-0-mm-3D_glszm_LargeAreaHighGrayLevelEmphasis | | | | | |
| 45 | log-sigma-3-0-mm-3D_glszm_SizeZoneNonUniformityNormalized | | | | | |
| 46 | log-sigma-4-0-mm-3D_firstorder_Kurtosis | | | |  |  |
| 47 | log-sigma-4-0-mm-3D_firstorder_Maximum | | | | |  |
| 48 | log-sigma-4-0-mm-3D_firstorder_Mean | | | |  |  |
| 49 | log-sigma-4-0-mm-3D_firstorder_Skewness | | | | |  |
| 50 | log-sigma-4-0-mm-3D_firstorder_Variance | | | | |  |
| 51 | log-sigma-4-0-mm-3D_glcm_Autocorrelation | | | | |  |
| 52 | log-sigma-4-0-mm-3D_glcm_ClusterShade | | | | |  |
| 53 | log-sigma-4-0-mm-3D_glcm_Correlation | | | |  |  |
| 54 | log-sigma-4-0-mm-3D_glcm_Idmn | | | |  |  |
| 55 | log-sigma-4-0-mm-3D_glcm_Imc1 | | | |  |  |
| 56 | log-sigma-4-0-mm-3D_glcm_JointEnergy | | | |  |  |
| 57 | log-sigma-4-0-mm-3D_gldm_DependenceNonUniformityNormalized | | | | | |
| 58 | log-sigma-4-0-mm-3D_gldm_DependenceVariance | | | | |  |
| 59 | log-sigma-4-0-mm-3D_gldm_LargeDependenceLowGrayLevelEmphasis | | | | | |
| 60 | log-sigma-4-0-mm-3D_gldm_SmallDependenceHighGrayLevelEmphasis | | | | | |
| 61 | log-sigma-4-0-mm-3D_gldm_SmallDependenceLowGrayLevelEmphasis | | | | | |
| 62 | log-sigma-4-0-mm-3D_glszm_LargeAreaEmphasis | | | | |  |
| 63 | log-sigma-4-0-mm-3D_glszm_LargeAreaLowGrayLevelEmphasis | | | | | |
| 64 | log-sigma-4-0-mm-3D_glszm_SizeZoneNonUniformityNormalized | | | | | |
| 65 | log-sigma-4-0-mm-3D_glszm_ZoneEntropy | | | | |  |
| 66 | log-sigma-5-0-mm-3D_firstorder_90Percentile | | | | |  |
| 67 | log-sigma-5-0-mm-3D_firstorder_Entropy | | | |  |  |
| 68 | log-sigma-5-0-mm-3D_firstorder_InterquartileRange | | | | |  |
| 69 | log-sigma-5-0-mm-3D_firstorder_Kurtosis | | | |  |  |
| 70 | log-sigma-5-0-mm-3D_firstorder_Maximum | | | | |  |
| 71 | log-sigma-5-0-mm-3D_firstorder_Minimum | | | | |  |
| 72 | log-sigma-5-0-mm-3D_firstorder_Range | | | |  |  |
| 73 | log-sigma-5-0-mm-3D_firstorder_Skewness | | | | |  |
| 74 | log-sigma-5-0-mm-3D_glcm_Autocorrelation | | | | |  |
| 75 | log-sigma-5-0-mm-3D_glcm_ClusterProminence | | | | |  |
| 76 | log-sigma-5-0-mm-3D_glcm_ClusterShade | | | | |  |
| 77 | log-sigma-5-0-mm-3D_glcm_Contrast | | | |  |  |
| 78 | log-sigma-5-0-mm-3D_glcm_Correlation | | | |  |  |
| 79 | log-sigma-5-0-mm-3D_glcm_Idmn | | | |  |  |
| 80 | log-sigma-5-0-mm-3D_glcm_Imc1 | | | |  |  |
| 81 | log-sigma-5-0-mm-3D_glcm_Imc2 | | | |  |  |
| 82 | log-sigma-5-0-mm-3D_glcm_InverseVariance | | | | |  |
| 83 | log-sigma-5-0-mm-3D_gldm_DependenceVariance | | | | |  |
| 84 | log-sigma-5-0-mm-3D_gldm_LargeDependenceHighGrayLevelEmphasis | | | | | |
| 85 | log-sigma-5-0-mm-3D_gldm_LargeDependenceLowGrayLevelEmphasis | | | | | |
| 86 | log-sigma-5-0-mm-3D_gldm_SmallDependenceLowGrayLevelEmphasis | | | | | |
| 87 | log-sigma-5-0-mm-3D_glszm_LargeAreaHighGrayLevelEmphasis | | | | | |
| 88 | log-sigma-5-0-mm-3D_glszm_LowGrayLevelZoneEmphasis | | | | | |
| 89 | log-sigma-5-0-mm-3D_glszm_SizeZoneNonUniformityNormalized | | | | | |
| 90 | log-sigma-5-0-mm-3D_glszm_SmallAreaLowGrayLevelEmphasis | | | | | |
| 91 | original_firstorder_10Percentile | | |  |  |  |
| 92 | original_firstorder_90Percentile | | |  |  |  |
| 93 | original_firstorder_Entropy | | |  |  |  |
| 94 | original_firstorder_Kurtosis | | |  |  |  |
| 95 | original_firstorder_Maximum | | |  |  |  |
| 96 | original_firstorder_Minimum | | |  |  |  |
| 97 | original_firstorder_Range | | |  |  |  |
| 98 | original_firstorder_Skewness | | |  |  |  |
| 99 | original_glcm_ClusterProminence | | | |  |  |
| 100 | original_glcm_ClusterShade | | |  |  |  |
| 101 | original_glcm_Contrast | | |  |  |  |
| 102 | original_glcm_Correlation | | |  |  |  |
| 103 | original_glcm_Id | |  |  |  |  |
| 104 | original_glcm_Idmn | |  |  |  |  |
| 105 | original_glcm_Imc2 | |  |  |  |  |
| 106 | original_glcm_JointEnergy | | |  |  |  |
| 107 | original_gldm_LargeDependenceHighGrayLevelEmphasis | | | | | |
| 108 | original_gldm_LargeDependenceLowGrayLevelEmphasis | | | | | |
| 109 | original_gldm_LowGrayLevelEmphasis | | | |  |  |
| 110 | original_gldm_SmallDependenceHighGrayLevelEmphasis | | | | | |
| 111 | original_glszm_LargeAreaEmphasis | | | |  |  |
| 112 | original_glszm_SizeZoneNonUniformityNormalized | | | | |  |
| 113 | original_shape_Elongation | | |  |  |  |
| 114 | original_shape_Flatness | | |  |  |  |
| 115 | original_shape_LeastAxisLength | | |  |  |  |
| 116 | original_shape_MajorAxisLength | | |  |  |  |
| 117 | original_shape_Maximum2DDiameterColumn | | | | |  |
| 118 | original_shape_Maximum2DDiameterRow | | | |  |  |
| 119 | original_shape_MinorAxisLength | | |  |  |  |
| 120 | original_shape_Sphericity | | |  |  |  |
| 121 | original_shape_SurfaceVolumeRatio | | | |  |  |
| 122 | wavelet-HHH_firstorder_10Percentile | | | |  |  |
| 123 | wavelet-HHH_firstorder_Kurtosis | | |  |  |  |
| 124 | wavelet-HHH_firstorder_Maximum | | | |  |  |
| 125 | wavelet-HHH_firstorder_Mean | | |  |  |  |
| 126 | wavelet-HHH_firstorder_Median | | |  |  |  |
| 127 | wavelet-HHH_firstorder_Minimum | | | |  |  |
| 128 | wavelet-HHH_firstorder_Skewness | | | |  |  |
| 129 | wavelet-HHH_glcm_ClusterShade | | | |  |  |
| 130 | wavelet-HHH_glcm_Correlation | | |  |  |  |
| 131 | wavelet-HHH_glcm_Idmn | | |  |  |  |
| 132 | wavelet-HHH_glcm_Imc1 | | |  |  |  |
| 133 | wavelet-HHH_glcm_Imc2 | | |  |  |  |
| 134 | wavelet-HHH_gldm_DependenceEntropy | | | |  |  |
| 135 | wavelet-HHH_gldm_DependenceNonUniformityNormalized | | | | | |
| 136 | wavelet-HHH_gldm_LargeDependenceHighGrayLevelEmphasis | | | | | |
| 137 | wavelet-HHH_gldm_LargeDependenceLowGrayLevelEmphasis | | | | | |
| 138 | wavelet-HHH_gldm_SmallDependenceHighGrayLevelEmphasis | | | | | |
| 139 | wavelet-HHH_gldm_SmallDependenceLowGrayLevelEmphasis | | | | | |
| 140 | wavelet-HHH_glrlm_LongRunEmphasis | | | |  |  |
| 141 | wavelet-HHH_glrlm_ShortRunLowGrayLevelEmphasis | | | | |  |
| 142 | wavelet-HHH_glszm_GrayLevelNonUniformityNormalized | | | | | |
| 143 | wavelet-HHH_glszm_LargeAreaEmphasis | | | |  |  |
| 144 | wavelet-HHH_glszm_LargeAreaHighGrayLevelEmphasis | | | | | |
| 145 | wavelet-HHH_glszm_LargeAreaLowGrayLevelEmphasis | | | | |  |
| 146 | wavelet-HHH_glszm_SizeZoneNonUniformity | | | | |  |
| 147 | wavelet-HHH_glszm_SizeZoneNonUniformityNormalized | | | | | |
| 148 | wavelet-HHH_glszm_ZonePercentage | | | |  |  |
| 149 | wavelet-HHL_firstorder_10Percentile | | | |  |  |
| 150 | wavelet-HHL_firstorder_Kurtosis | | |  |  |  |
| 151 | wavelet-HHL_firstorder_Maximum | | | |  |  |
| 152 | wavelet-HHL_firstorder_Mean | | |  |  |  |
| 153 | wavelet-HHL_firstorder_Median | | |  |  |  |
| 154 | wavelet-HHL_firstorder_Minimum | | | |  |  |
| 155 | wavelet-HHL_firstorder_RootMeanSquared | | | |  |  |
| 156 | wavelet-HHL_firstorder_Skewness | | | |  |  |
| 157 | wavelet-HHL_glcm_ClusterProminence | | | |  |  |
| 158 | wavelet-HHL_glcm_ClusterShade | | | |  |  |
| 159 | wavelet-HHL_glcm_Correlation | | |  |  |  |
| 160 | wavelet-HHL_glcm_Idmn | | |  |  |  |
| 161 | wavelet-HHL_glcm_Imc1 | | |  |  |  |
| 162 | wavelet-HHL_glcm_JointEnergy | | |  |  |  |
| 163 | wavelet-HHL_gldm_DependenceEntropy | | | |  |  |
| 164 | wavelet-HHL_gldm_DependenceVariance | | | |  |  |
| 165 | wavelet-HHL_gldm_LargeDependenceHighGrayLevelEmphasis | | | | | |
| 166 | wavelet-HHL_gldm_LargeDependenceLowGrayLevelEmphasis | | | | | |
| 167 | wavelet-HHL_gldm_SmallDependenceLowGrayLevelEmphasis | | | | | |
| 168 | wavelet-HHL_glszm_LargeAreaEmphasis | | | |  |  |
| 169 | wavelet-HHL_glszm_LargeAreaHighGrayLevelEmphasis | | | | | |
| 170 | wavelet-HHL_glszm_SizeZoneNonUniformityNormalized | | | | | |
| 171 | wavelet-HLH_firstorder_Kurtosis | | |  |  |  |
| 172 | wavelet-HLH_firstorder_Maximum | | | |  |  |
| 173 | wavelet-HLH_firstorder_Mean | | |  |  |  |
| 174 | wavelet-HLH_firstorder_Median | | |  |  |  |
| 175 | wavelet-HLH_firstorder_Minimum | | | |  |  |
| 176 | wavelet-HLH_firstorder_Skewness | | | |  |  |
| 177 | wavelet-HLH_firstorder_Variance | | |  |  |  |
| 178 | wavelet-HLH_glcm_ClusterProminence | | | |  |  |
| 179 | wavelet-HLH_glcm_ClusterShade | | | |  |  |
| 180 | wavelet-HLH_glcm_Correlation | | |  |  |  |
| 181 | wavelet-HLH_glcm_Idmn | | |  |  |  |
| 182 | wavelet-HLH_glcm_Imc1 | | |  |  |  |
| 183 | wavelet-HLH_glcm_Imc2 | | |  |  |  |
| 184 | wavelet-HLH_glcm_JointEnergy | | |  |  |  |
| 185 | wavelet-HLH_gldm_DependenceEntropy | | | |  |  |
| 186 | wavelet-HLH_gldm_LargeDependenceHighGrayLevelEmphasis | | | | | |
| 187 | wavelet-HLH_gldm_LowGrayLevelEmphasis | | | |  |  |
| 188 | wavelet-HLH_gldm_SmallDependenceLowGrayLevelEmphasis | | | | | |
| 189 | wavelet-HLH_glszm_LargeAreaHighGrayLevelEmphasis | | | | | |
| 190 | wavelet-HLH_glszm_SizeZoneNonUniformityNormalized | | | | | |
| 191 | wavelet-HLH_glszm_ZoneEntropy | | | |  |  |
| 192 | wavelet-HLL_firstorder_10Percentile | | | |  |  |
| 193 | wavelet-HLL_firstorder_90Percentile | | | |  |  |
| 194 | wavelet-HLL_firstorder_Kurtosis | | |  |  |  |
| 195 | wavelet-HLL_firstorder_Maximum | | | |  |  |
| 196 | wavelet-HLL_firstorder_Mean | | |  |  |  |
| 197 | wavelet-HLL_firstorder_Minimum | | | |  |  |
| 198 | wavelet-HLL_firstorder_RootMeanSquared | | | |  |  |
| 199 | wavelet-HLL_firstorder_Skewness | | | |  |  |
| 200 | wavelet-HLL_glcm_ClusterProminence | | | |  |  |
| 201 | wavelet-HLL_glcm_ClusterShade | | |  |  |  |
| 202 | wavelet-HLL_glcm_Correlation | | |  |  |  |
| 203 | wavelet-HLL_glcm_Id | |  |  |  |  |
| 204 | wavelet-HLL_glcm_Idmn | | |  |  |  |
| 205 | wavelet-HLL_glcm_Imc1 | | |  |  |  |
| 206 | wavelet-HLL_glcm_Imc2 | | |  |  |  |
| 207 | wavelet-HLL_glcm_JointEnergy | | |  |  |  |
| 208 | wavelet-HLL_gldm_DependenceEntropy | | | |  |  |
| 209 | wavelet-HLL_gldm_DependenceVariance | | | |  |  |
| 210 | wavelet-HLL_gldm_LargeDependenceHighGrayLevelEmphasis | | | | | |
| 211 | wavelet-HLL_gldm_LargeDependenceLowGrayLevelEmphasis | | | | | |
| 212 | wavelet-HLL_gldm_LowGrayLevelEmphasis | | | |  |  |
| 213 | wavelet-HLL_gldm_SmallDependenceLowGrayLevelEmphasis | | | | | |
| 214 | wavelet-HLL_glszm_GrayLevelNonUniformityNormalized | | | | | |
| 215 | wavelet-HLL_glszm_LargeAreaHighGrayLevelEmphasis | | | | |  |
| 216 | wavelet-HLL_glszm_SizeZoneNonUniformityNormalized | | | | | |
| 217 | wavelet-LHH_firstorder_90Percentile | | | |  |  |
| 218 | wavelet-LHH_firstorder_Kurtosis | | |  |  |  |
| 219 | wavelet-LHH_firstorder_Maximum | | | |  |  |
| 220 | wavelet-LHH_firstorder_Mean | | |  |  |  |
| 221 | wavelet-LHH_firstorder_Median | | |  |  |  |
| 222 | wavelet-LHH_firstorder_Minimum | | | |  |  |
| 223 | wavelet-LHH_firstorder_Skewness | | | |  |  |
| 224 | wavelet-LHH_glcm_ClusterProminence | | | |  |  |
| 225 | wavelet-LHH_glcm_ClusterShade | | | |  |  |
| 226 | wavelet-LHH_glcm_Correlation | | |  |  |  |
| 227 | wavelet-LHH_glcm_Idmn | | |  |  |  |
| 228 | wavelet-LHH_glcm_Imc1 | | |  |  |  |
| 229 | wavelet-LHH_glcm_Imc2 | | |  |  |  |
| 230 | wavelet-LHH_glcm_MaximumProbability | | | |  |  |
| 231 | wavelet-LHH_gldm_DependenceEntropy | | | |  |  |
| 232 | wavelet-LHH_gldm_LargeDependenceHighGrayLevelEmphasis | | | | | |
| 233 | wavelet-LHH_gldm_LowGrayLevelEmphasis | | | |  |  |
| 234 | wavelet-LHH_gldm_SmallDependenceHighGrayLevelEmphasis | | | | | |
| 235 | wavelet-LHH_gldm_SmallDependenceLowGrayLevelEmphasis | | | | | |
| 236 | wavelet-LHH_glrlm_RunEntropy | | |  |  |  |
| 237 | wavelet-LHH_glszm_SizeZoneNonUniformityNormalized | | | | | |
| 238 | wavelet-LHL_firstorder_10Percentile | | | |  |  |
| 239 | wavelet-LHL_firstorder_90Percentile | | | |  |  |
| 240 | wavelet-LHL_firstorder_Kurtosis | | |  |  |  |
| 241 | wavelet-LHL_firstorder_Maximum | | | |  |  |
| 242 | wavelet-LHL_firstorder_Mean | | |  |  |  |
| 243 | wavelet-LHL_firstorder_Minimum | | | |  |  |
| 244 | wavelet-LHL_firstorder_RootMeanSquared | | | |  |  |
| 245 | wavelet-LHL_firstorder_Skewness | | | |  |  |
| 246 | wavelet-LHL_firstorder_Uniformity | | | |  |  |
| 247 | wavelet-LHL_glcm_ClusterShade | | |  |  |  |
| 248 | wavelet-LHL_glcm_Correlation | | |  |  |  |
| 249 | wavelet-LHL_glcm_DifferenceVariance | | | |  |  |
| 250 | wavelet-LHL_glcm_Idmn | | |  |  |  |
| 251 | wavelet-LHL_glcm_Imc2 | | |  |  |  |
| 252 | wavelet-LHL_glcm_JointEnergy | | |  |  |  |
| 253 | wavelet-LHL_glcm_JointEntropy | | |  |  |  |
| 254 | wavelet-LHL_gldm_DependenceNonUniformityNormalized | | | | | |
| 255 | wavelet-LHL_gldm_DependenceVariance | | | |  |  |
| 256 | wavelet-LHL_gldm_LargeDependenceHighGrayLevelEmphasis | | | | | |
| 257 | wavelet-LHL_gldm_LargeDependenceLowGrayLevelEmphasis | | | | | |
| 258 | wavelet-LHL_gldm_LowGrayLevelEmphasis | | | |  |  |
| 259 | wavelet-LHL_glszm_LargeAreaEmphasis | | | |  |  |
| 260 | wavelet-LHL_glszm_LargeAreaHighGrayLevelEmphasis | | | | |  |
| 261 | wavelet-LLH_firstorder_10Percentile | | | |  |  |
| 262 | wavelet-LLH_firstorder_90Percentile | | | |  |  |
| 263 | wavelet-LLH_firstorder_Entropy | | |  |  |  |
| 264 | wavelet-LLH_firstorder_Kurtosis | | |  |  |  |
| 265 | wavelet-LLH_firstorder_Maximum | | | |  |  |
| 266 | wavelet-LLH_firstorder_Mean | | |  |  |  |
| 267 | wavelet-LLH_firstorder_Minimum | | | |  |  |
| 268 | wavelet-LLH_firstorder_RootMeanSquared | | | |  |  |
| 269 | wavelet-LLH_firstorder_Skewness | | | |  |  |
| 270 | wavelet-LLH_glcm_Correlation | | |  |  |  |
| 271 | wavelet-LLH_glcm_Idmn | | |  |  |  |
| 272 | wavelet-LLH_glcm_Idn | | |  |  |  |
| 273 | wavelet-LLH_glcm_Imc1 | | |  |  |  |
| 274 | wavelet-LLH_glcm_Imc2 | | |  |  |  |
| 275 | wavelet-LLH_glcm_JointEnergy | | |  |  |  |
| 276 | wavelet-LLH_gldm_DependenceEntropy | | | |  |  |
| 277 | wavelet-LLH_gldm_DependenceNonUniformityNormalized | | | | | |
| 278 | wavelet-LLH_gldm_DependenceVariance | | | |  |  |
| 279 | wavelet-LLH_gldm_LargeDependenceHighGrayLevelEmphasis | | | | | |
| 280 | wavelet-LLH_gldm_LargeDependenceLowGrayLevelEmphasis | | | | | |
| 281 | wavelet-LLH_gldm_LowGrayLevelEmphasis | | | |  |  |
| 282 | wavelet-LLH_gldm_SmallDependenceLowGrayLevelEmphasis | | | | | |
| 283 | wavelet-LLH_glszm_SizeZoneNonUniformityNormalized | | | | | |
| 284 | wavelet-LLH_glszm_SmallAreaLowGrayLevelEmphasis | | | | |  |
| 285 | wavelet-LLH_glszm_ZoneEntropy | | | |  |  |
| 286 | wavelet-LLL_glcm_JointEntropy | | |  |  |  |
| 287 | wavelet-LLL_gldm_LargeDependenceEmphasis | | | | |  |
| 288 | wavelet-LLL_gldm_LargeDependenceLowGrayLevelEmphasis | | | | | |
| 289 | wavelet-LLL_gldm_LowGrayLevelEmphasis | | | |  |  |
| 290 | wavelet-LLL_glszm_LargeAreaHighGrayLevelEmphasis | | | | |  |
| 291 | wavelet-LLL_glszm_ZoneEntropy | | | |  |  |

|  | ***second selected*** |  |  |  |
| --- | --- | --- | --- | --- |
| 0 | log-sigma-2-0-mm-3D_firstorder_Entropy | | |  |
| 1 | log-sigma-2-0-mm-3D_firstorder_Maximum | | | |
| 2 | log-sigma-2-0-mm-3D_firstorder_Mean | | |  |
| 3 | log-sigma-2-0-mm-3D_firstorder_Minimum | | | |
| 4 | log-sigma-2-0-mm-3D_firstorder_Range | | |  |
| 5 | log-sigma-2-0-mm-3D_glcm_ClusterProminence | | | |
| 6 | log-sigma-2-0-mm-3D_glcm_ClusterShade | | | |
| 7 | log-sigma-2-0-mm-3D_glcm_JointEnergy | | |  |
| 8 | log-sigma-2-0-mm-3D_glszm_SizeZoneNonUniformity | | | |
| 9 | log-sigma-3-0-mm-3D_firstorder_10Percentile | | | |
| 10 | log-sigma-3-0-mm-3D_firstorder_Maximum | | | |
| 11 | log-sigma-3-0-mm-3D_firstorder_Minimum | | | |
| 12 | log-sigma-3-0-mm-3D_firstorder_Range | | |  |
| 13 | log-sigma-3-0-mm-3D_firstorder_RobustMeanAbsoluteDeviation | | | |
| 14 | log-sigma-3-0-mm-3D_glcm_InverseVariance | | | |
| 15 | log-sigma-3-0-mm-3D_glrlm_RunEntropy | | |  |
| 16 | log-sigma-3-0-mm-3D_glszm_GrayLevelVariance | | | |
| 17 | log-sigma-3-0-mm-3D_glszm_SizeZoneNonUniformityNormalized | | | |
| 18 | log-sigma-4-0-mm-3D_firstorder_Maximum | | | |
| 19 | log-sigma-4-0-mm-3D_firstorder_Mean | | |  |
| 20 | log-sigma-4-0-mm-3D_firstorder_Variance | | | |
| 21 | log-sigma-4-0-mm-3D_glcm_Autocorrelation | | | |
| 22 | log-sigma-4-0-mm-3D_glcm_JointEnergy | | |  |
| 23 | log-sigma-4-0-mm-3D_gldm_DependenceNonUniformityNormalized | | | |
| 24 | log-sigma-4-0-mm-3D_gldm_DependenceVariance | | | |
| 25 | log-sigma-4-0-mm-3D_gldm_LargeDependenceLowGrayLevelEmphasis | | | |
| 26 | log-sigma-4-0-mm-3D_gldm_SmallDependenceHighGrayLevelEmphasis | | | |
| 27 | log-sigma-4-0-mm-3D_glszm_LargeAreaLowGrayLevelEmphasis | | | |
| 28 | log-sigma-4-0-mm-3D_glszm_SizeZoneNonUniformityNormalized | | | |
| 29 | log-sigma-4-0-mm-3D_glszm_ZoneEntropy | | | |
| 30 | log-sigma-5-0-mm-3D_firstorder_Entropy | | |  |
| 31 | log-sigma-5-0-mm-3D_firstorder_InterquartileRange | | | |
| 32 | log-sigma-5-0-mm-3D_firstorder_Maximum | | | |
| 33 | log-sigma-5-0-mm-3D_firstorder_Minimum | | | |
| 34 | log-sigma-5-0-mm-3D_firstorder_Range | | |  |
| 35 | log-sigma-5-0-mm-3D_glcm_Autocorrelation | | | |
| 36 | log-sigma-5-0-mm-3D_glcm_ClusterProminence | | | |
| 37 | log-sigma-5-0-mm-3D_glcm_ClusterShade | | | |
| 38 | log-sigma-5-0-mm-3D_glcm_Contrast | | |  |
| 39 | log-sigma-5-0-mm-3D_glcm_InverseVariance | | | |
| 40 | log-sigma-5-0-mm-3D_gldm_DependenceVariance | | | |
| 41 | log-sigma-5-0-mm-3D_gldm_LargeDependenceLowGrayLevelEmphasis | | | |
| 42 | log-sigma-5-0-mm-3D_glszm_LowGrayLevelZoneEmphasis | | | |
| 43 | log-sigma-5-0-mm-3D_glszm_SizeZoneNonUniformityNormalized | | | |
| 44 | log-sigma-5-0-mm-3D_glszm_SmallAreaLowGrayLevelEmphasis | | | |
| 45 | original_firstorder_90Percentile | |  |  |
| 46 | original_firstorder_Entropy | |  |  |
| 47 | original_firstorder_Maximum | |  |  |
| 48 | original_firstorder_Minimum | |  |  |
| 49 | original_firstorder_Range | |  |  |
| 50 | original_firstorder_Skewness | |  |  |
| 51 | original_glcm_ClusterProminence | | |  |
| 52 | original_glcm_ClusterShade | |  |  |
| 53 | original_glcm_Contrast | |  |  |
| 54 | original_glcm_Correlation | |  |  |
| 55 | original_glcm_Id |  |  |  |
| 56 | original_glcm_JointEnergy | |  |  |
| 57 | original_gldm_LargeDependenceHighGrayLevelEmphasis | | | |
| 58 | original_gldm_SmallDependenceHighGrayLevelEmphasis | | | |
| 59 | original_glszm_SizeZoneNonUniformityNormalized | | | |
| 60 | original_shape_Elongation | |  |  |
| 61 | original_shape_MajorAxisLength | |  |  |
| 62 | original_shape_Maximum2DDiameterRow | | |  |
| 63 | wavelet-HHH_firstorder_10Percentile | | |  |
| 64 | wavelet-HHH_firstorder_Maximum | | |  |
| 65 | wavelet-HHH_firstorder_Minimum | | |  |
| 66 | wavelet-HHH_gldm_DependenceEntropy | | |  |
| 67 | wavelet-HHH_gldm_SmallDependenceHighGrayLevelEmphasis | | | |
| 68 | wavelet-HHH_glszm_SizeZoneNonUniformity | | | |
| 69 | wavelet-HLH_firstorder_Maximum | | |  |
| 70 | wavelet-HLH_firstorder_Mean | |  |  |
| 71 | wavelet-HLH_firstorder_Median | |  |  |
| 72 | wavelet-HLH_firstorder_Minimum | | |  |
| 73 | wavelet-HLH_firstorder_Variance | |  |  |
| 74 | wavelet-HLH_glcm_ClusterProminence | | |  |
| 75 | wavelet-HLH_glcm_JointEnergy | |  |  |
| 76 | wavelet-HLH_gldm_DependenceEntropy | | |  |
| 77 | wavelet-HLH_gldm_LargeDependenceHighGrayLevelEmphasis | | | |
| 78 | wavelet-HLH_gldm_LowGrayLevelEmphasis | | |  |
| 79 | wavelet-HLH_glszm_SizeZoneNonUniformityNormalized | | | |
| 80 | wavelet-HLH_glszm_ZoneEntropy | | |  |
| 81 | wavelet-HLL_firstorder_Maximum | | |  |
| 82 | wavelet-HLL_gldm_LargeDependenceLowGrayLevelEmphasis | | | |
| 83 | wavelet-HLL_gldm_LowGrayLevelEmphasis | | |  |
| 84 | wavelet-LHH_firstorder_90Percentile | | |  |
| 85 | wavelet-LHH_firstorder_Minimum | | |  |
| 86 | wavelet-LHH_glcm_MaximumProbability | | |  |
| 87 | wavelet-LHH_gldm_DependenceEntropy | | |  |
| 88 | wavelet-LHH_gldm_LowGrayLevelEmphasis | | |  |
| 89 | wavelet-LHH_glrlm_RunEntropy | |  |  |
| 90 | wavelet-LHL_firstorder_Maximum | | |  |
| 91 | wavelet-LLH_firstorder_10Percentile | | |  |
| 92 | wavelet-LLH_firstorder_Entropy | |  |  |
| 93 | wavelet-LLH_firstorder_Maximum | | |  |
| 94 | wavelet-LLH_firstorder_Mean | |  |  |
| 95 | wavelet-LLH_firstorder_Minimum | | |  |
| 96 | wavelet-LLH_firstorder_RootMeanSquared | | |  |
| 97 | wavelet-LLH_glcm_Imc2 | |  |  |
| 98 | wavelet-LLH_glcm_JointEnergy | |  |  |
| 99 | wavelet-LLH_gldm_DependenceEntropy | | |  |
| 100 | wavelet-LLH_gldm_DependenceNonUniformityNormalized | | | |
| 101 | wavelet-LLH_gldm_LowGrayLevelEmphasis | | |  |
| 102 | wavelet-LLH_glszm_SizeZoneNonUniformityNormalized | | | |
| 103 | wavelet-LLH_glszm_ZoneEntropy | | |  |
| 104 | wavelet-LLL_gldm_LargeDependenceEmphasis | | | |
| 105 | wavelet-LLL_glszm_LargeAreaHighGrayLevelEmphasis | | | |
| 106 | wavelet-LLL_glszm_ZoneEntropy | | |  |

|  | ***Third selected*** |  |
| --- | --- | --- |
| 0 | original_glcm_ClusterShade | |
| 1 | original_glcm_Correlation | |
| 2 | original_gldm_LargeDependenceHighGrayLevelEmphasis | |
| 3 | original_shape_Maximum2DDiameterRow | |
| 4 | wavelet-HLH_glszm_SizeZoneNonUniformityNormalized | |
| 5 | wavelet-LLH_firstorder_10Percentile | |
| 6 | wavelet-LLH_firstorder_Mean | |
