# Supplementary figures and images for: Different MRI-based radiomics machine learning models to predict CD3+ tumor-infiltrating lymphocytes in rectal cancer
Source: Front Oncol. 2025 Apr 28;15:1509207. doi: 10.3389/fonc.2025.1509207 (PMC12066337; doi:10.3389/fonc.2025.1509207)

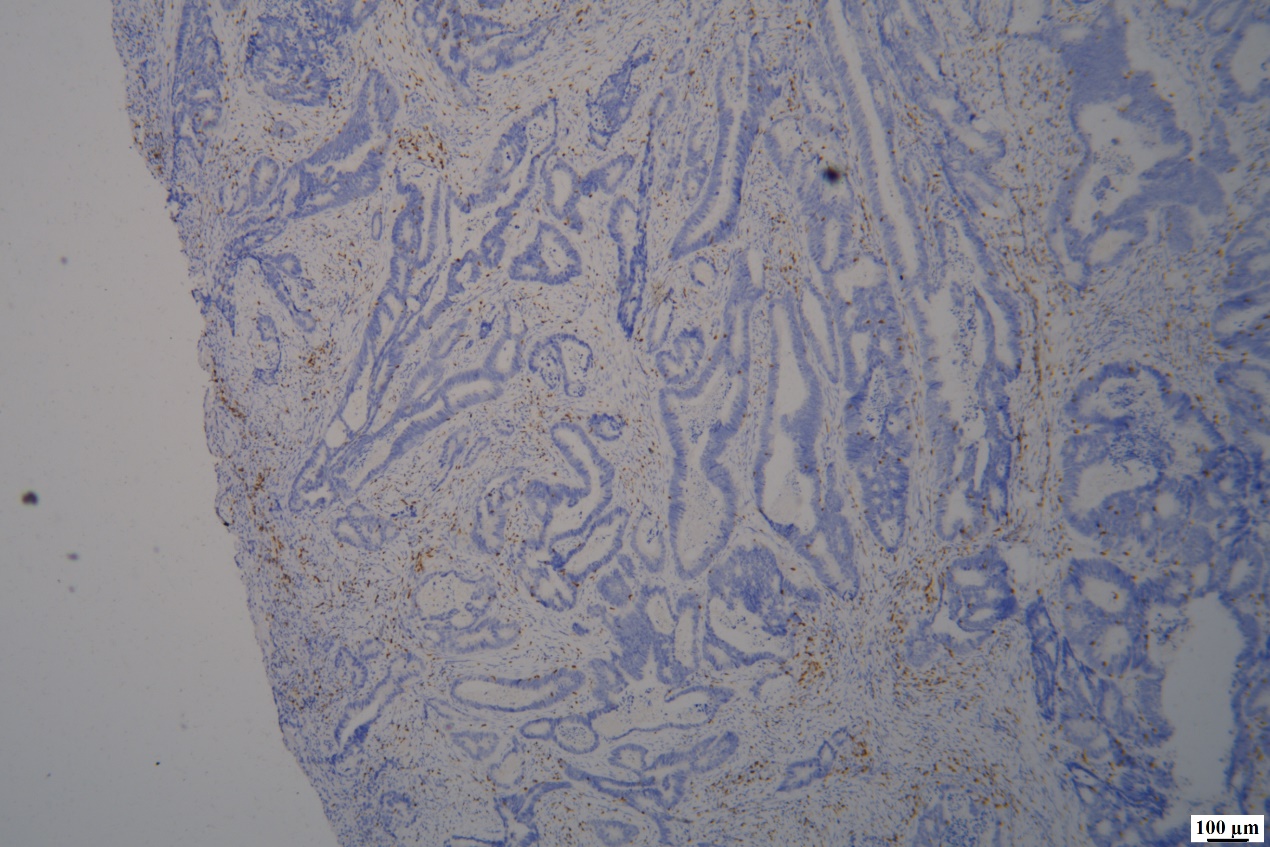

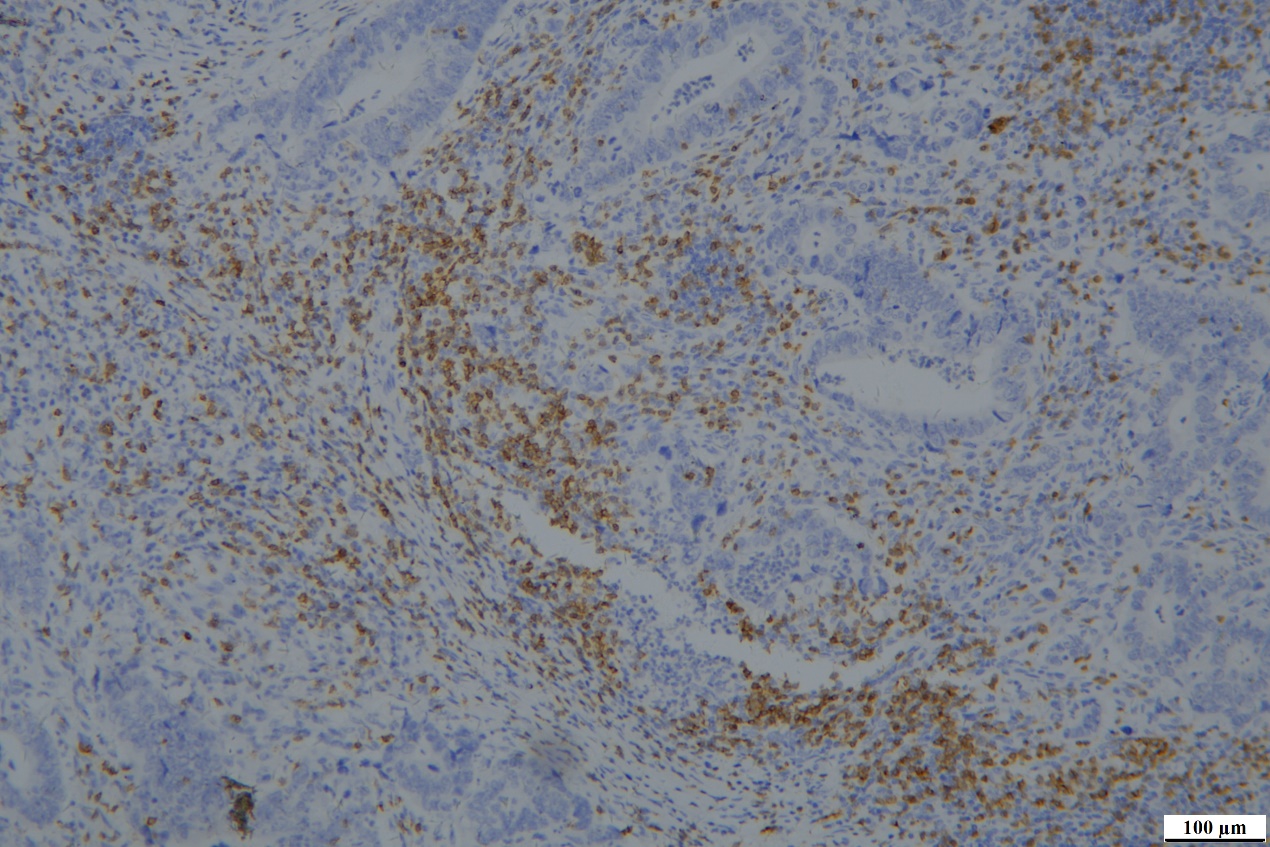

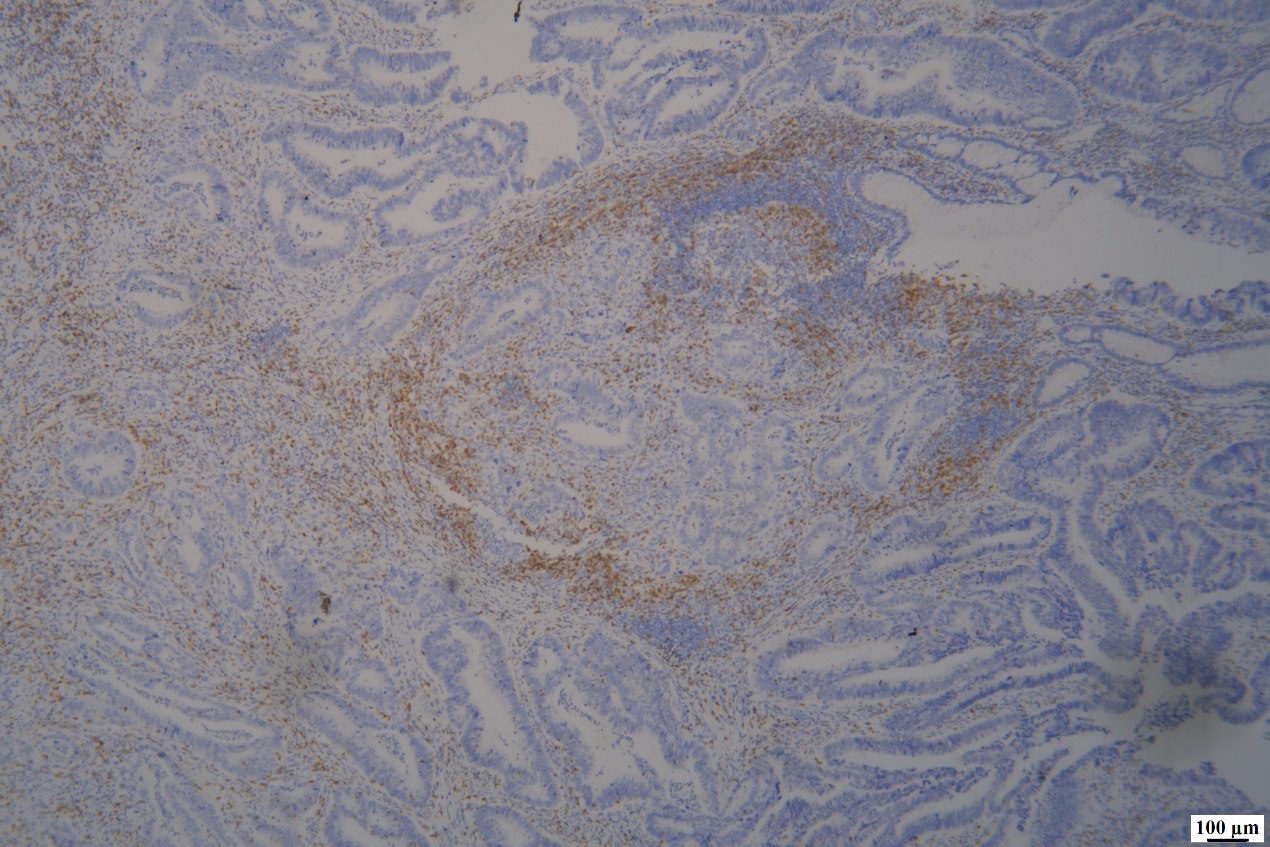

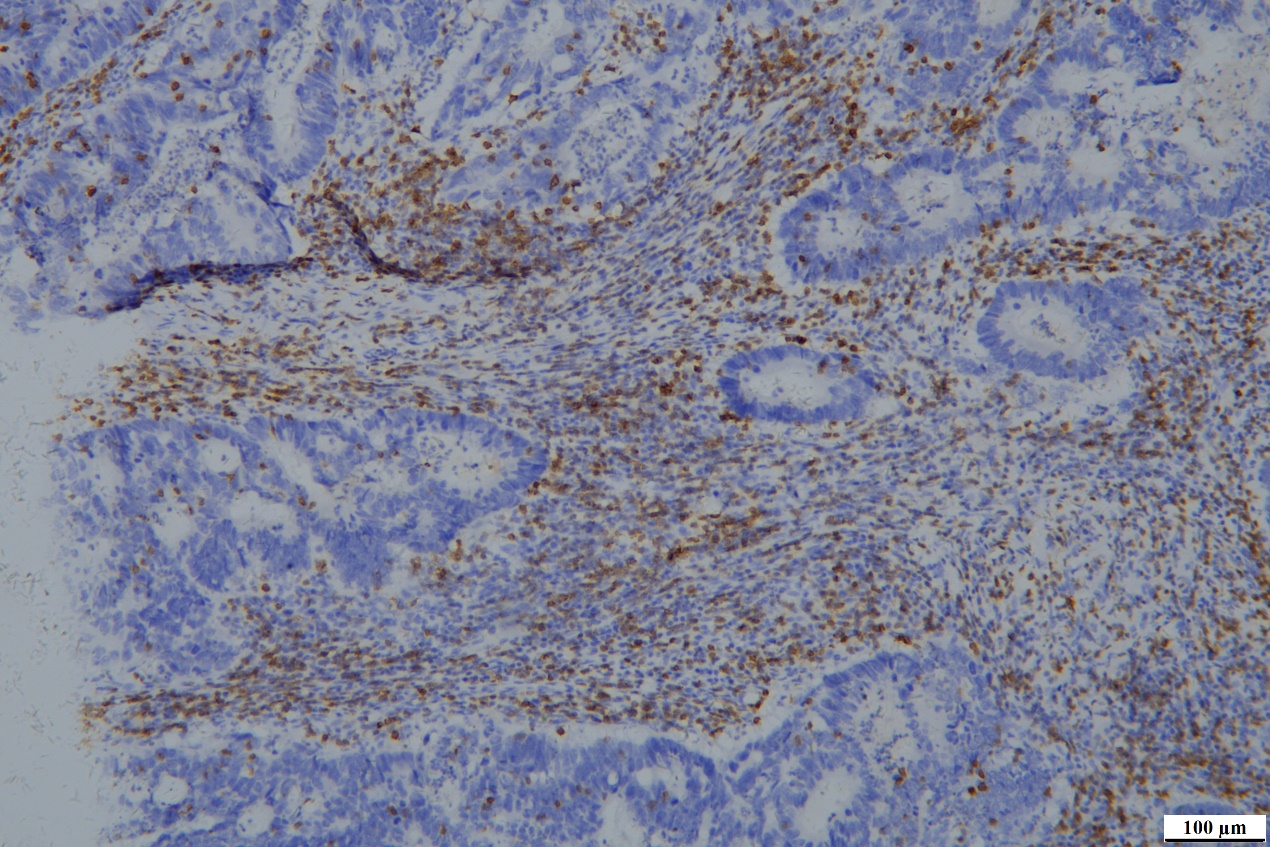

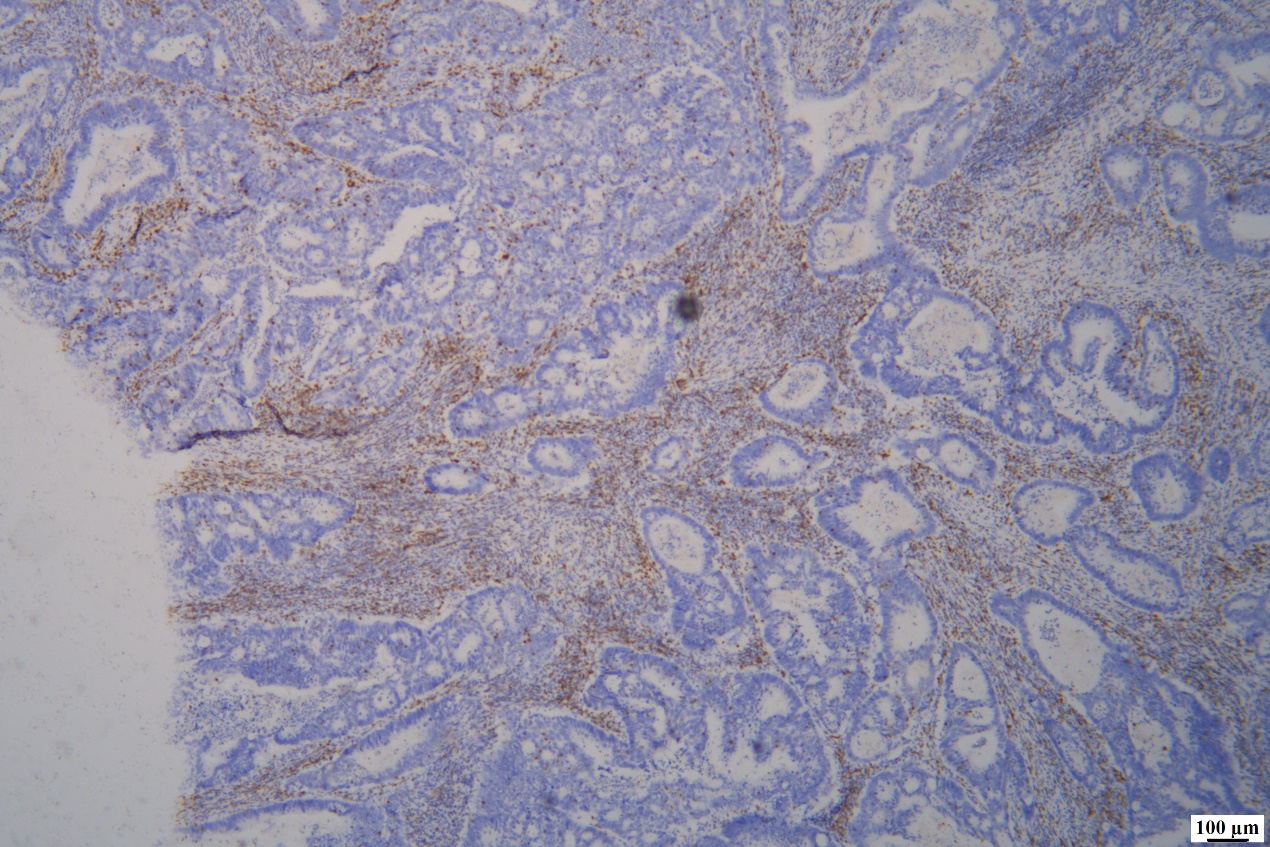

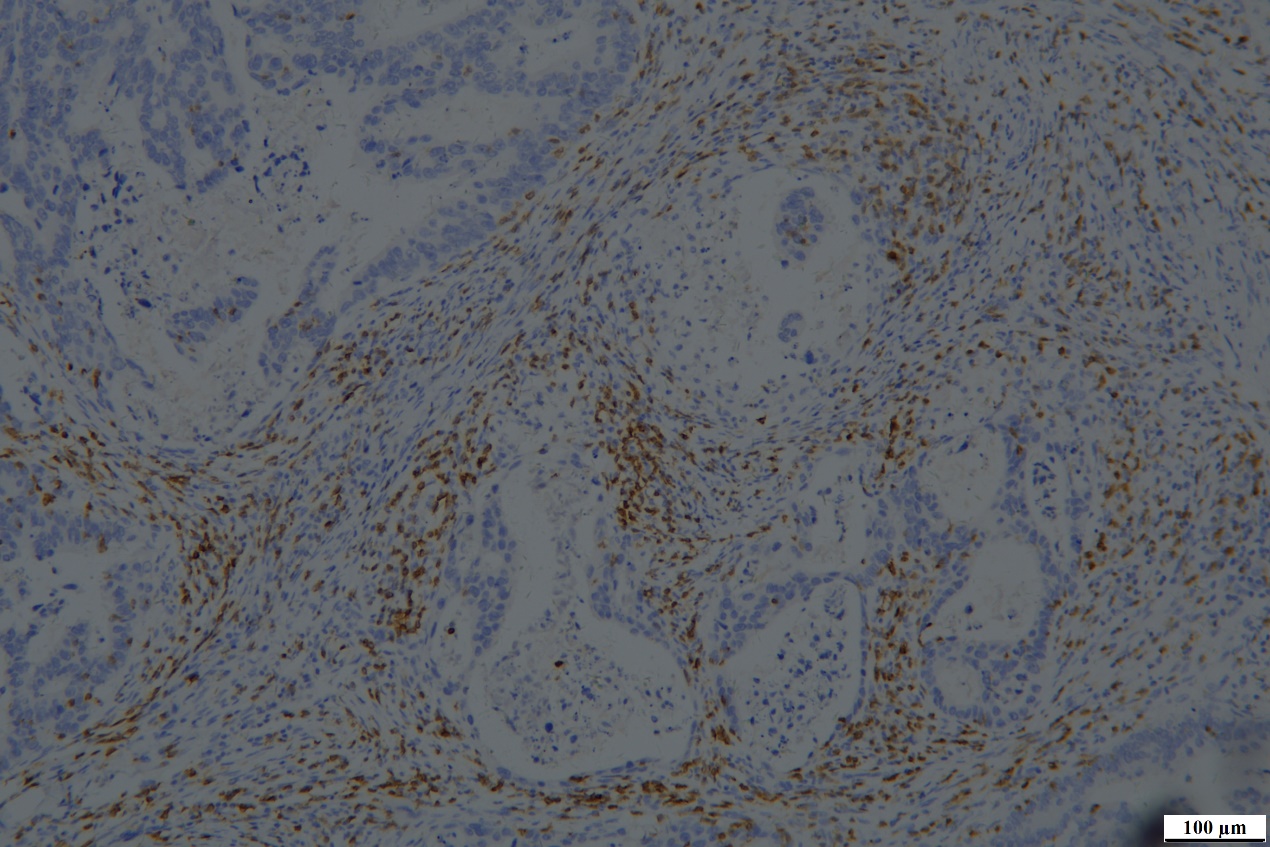

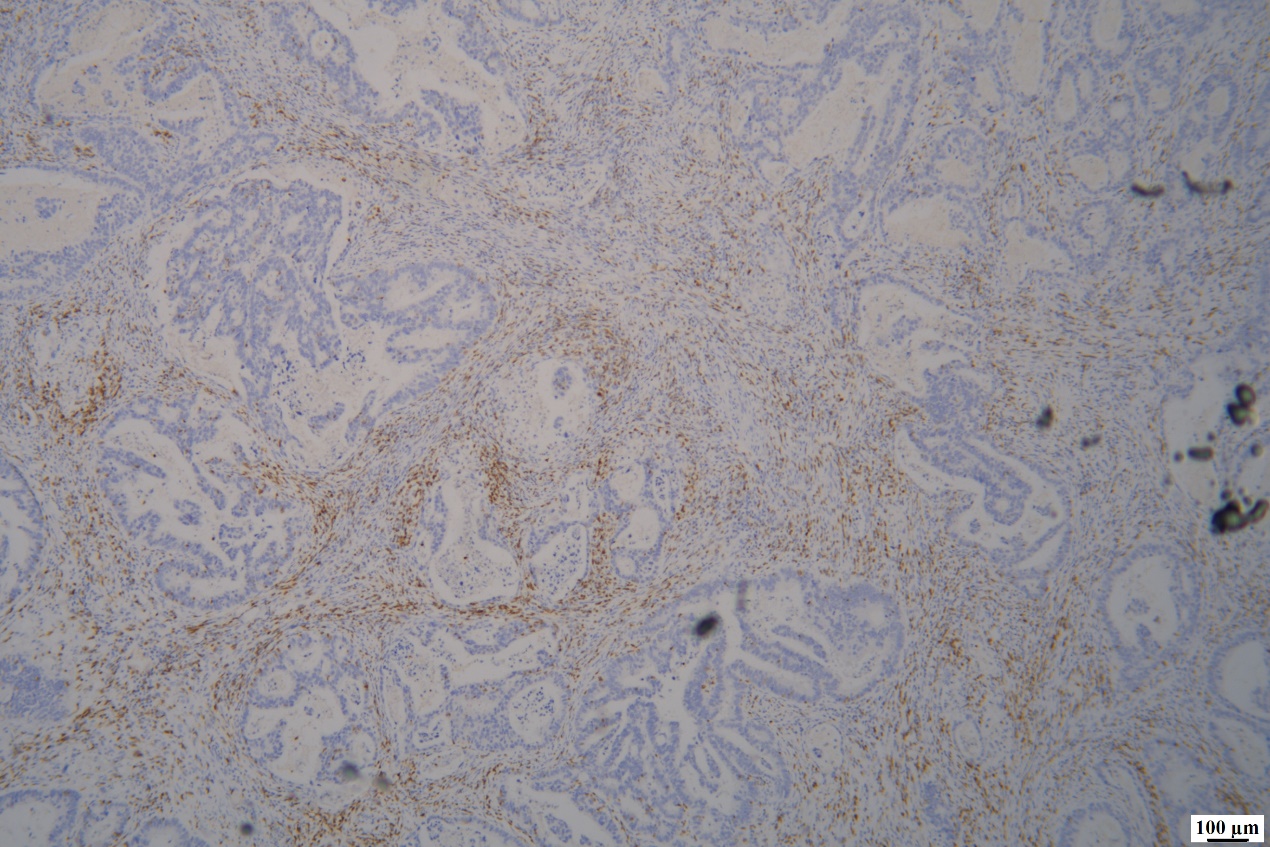

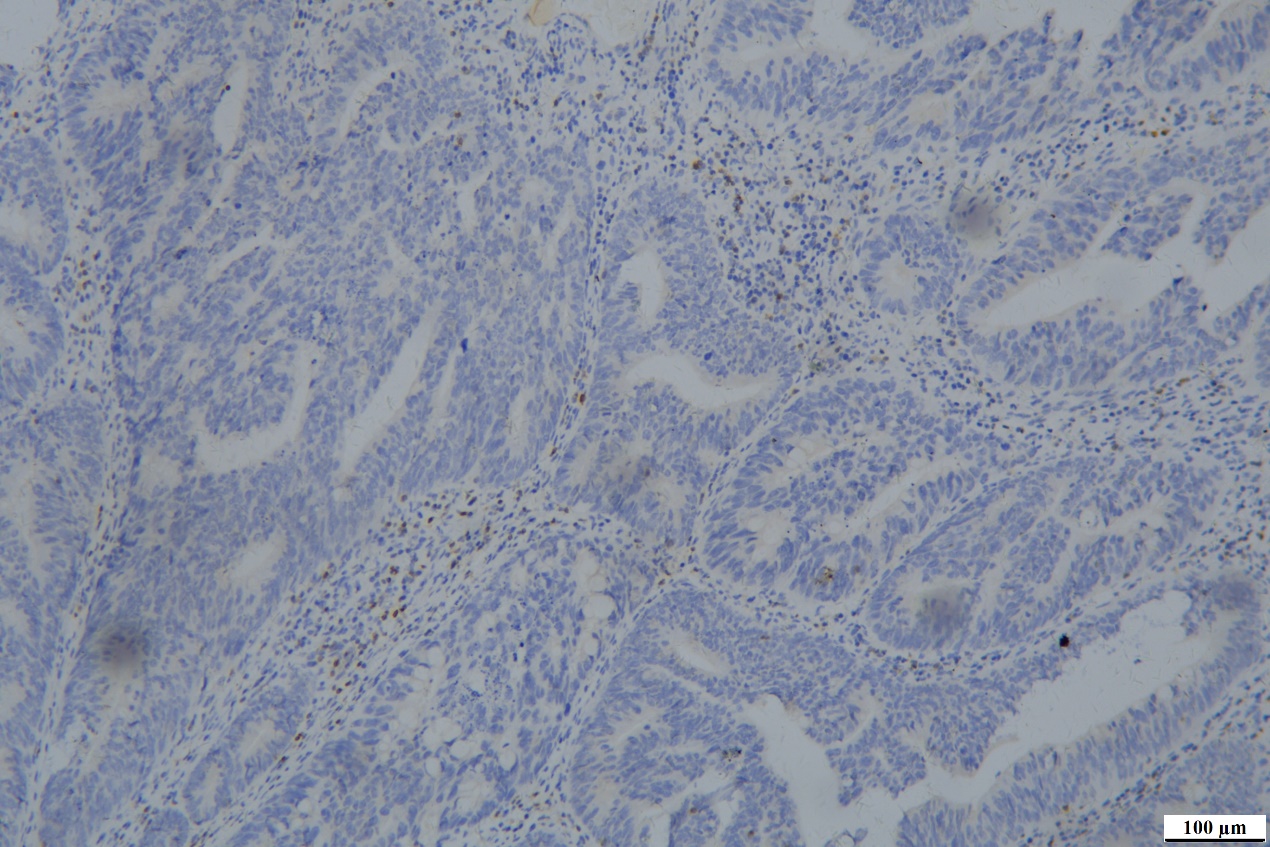

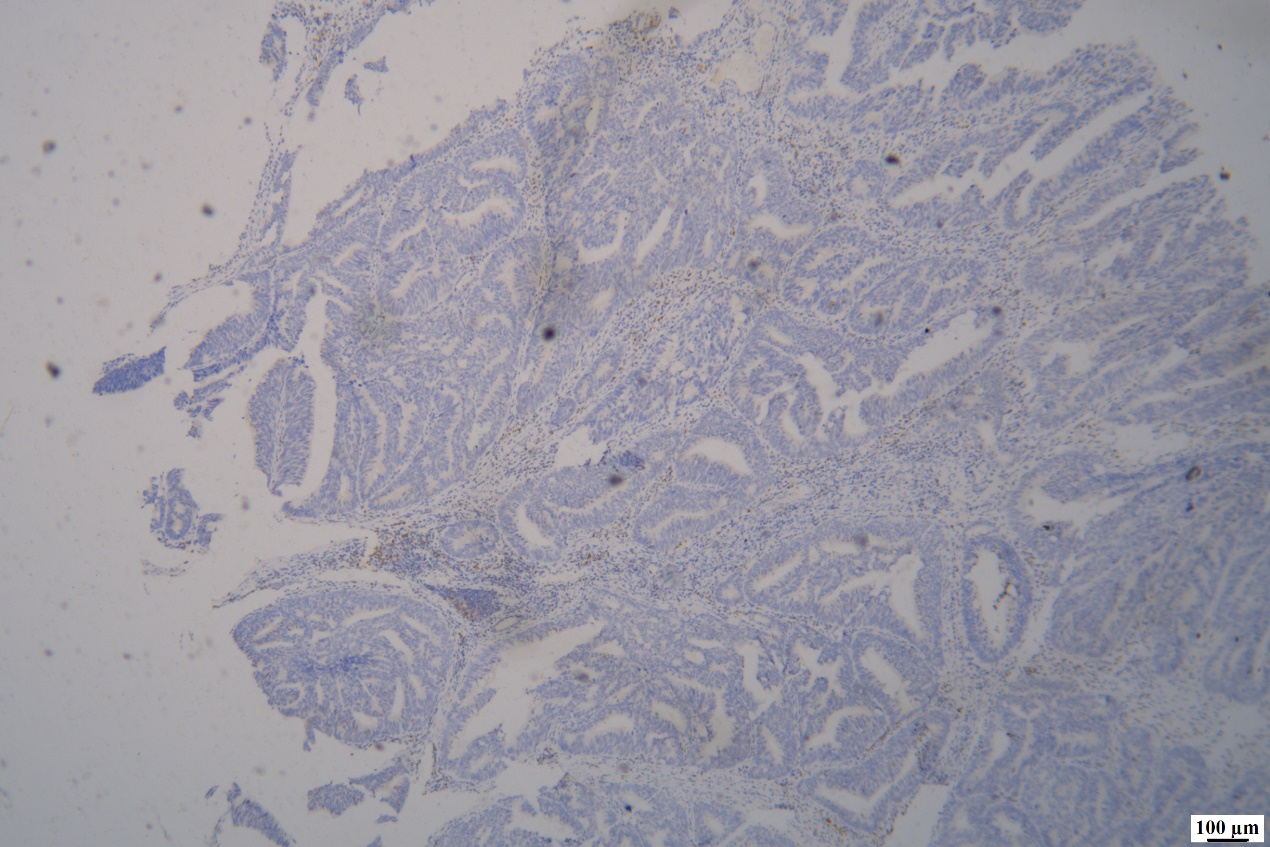

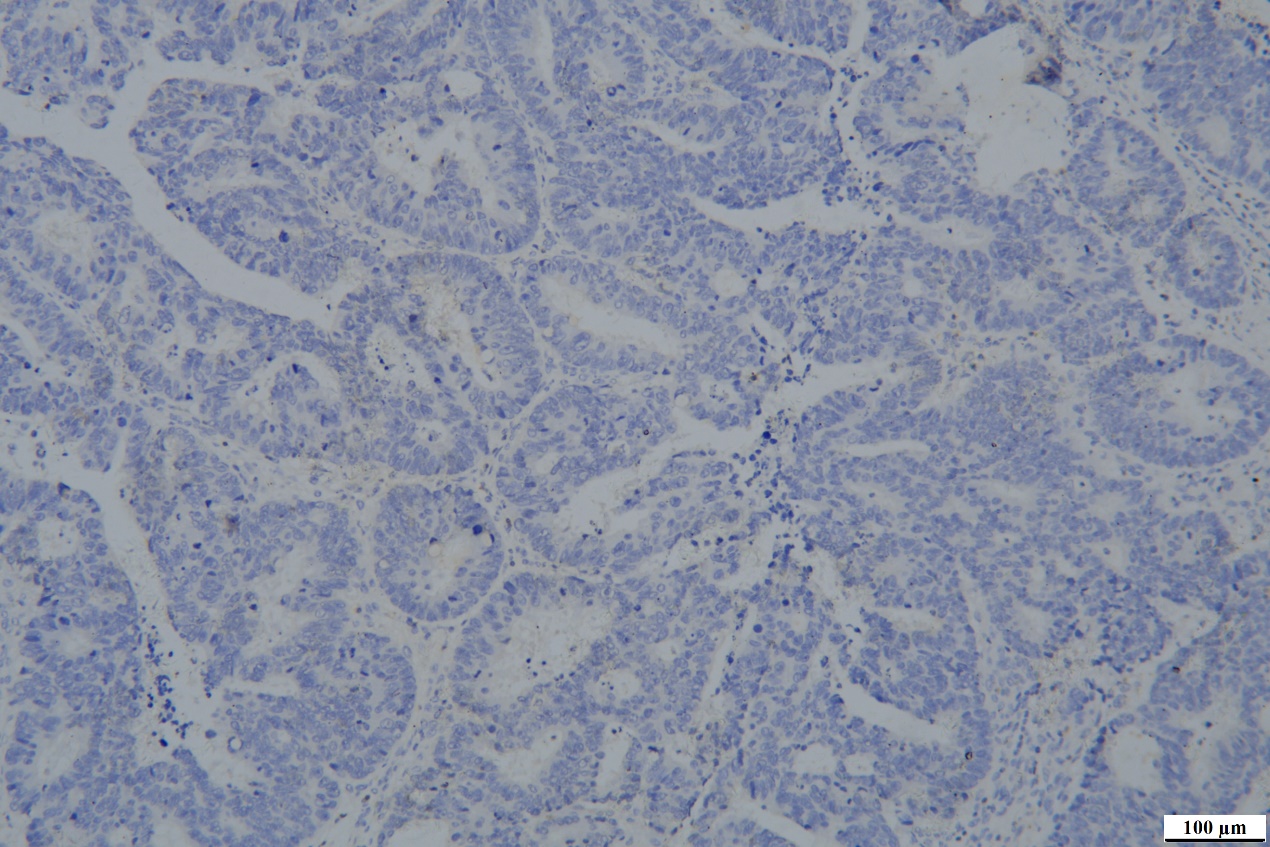

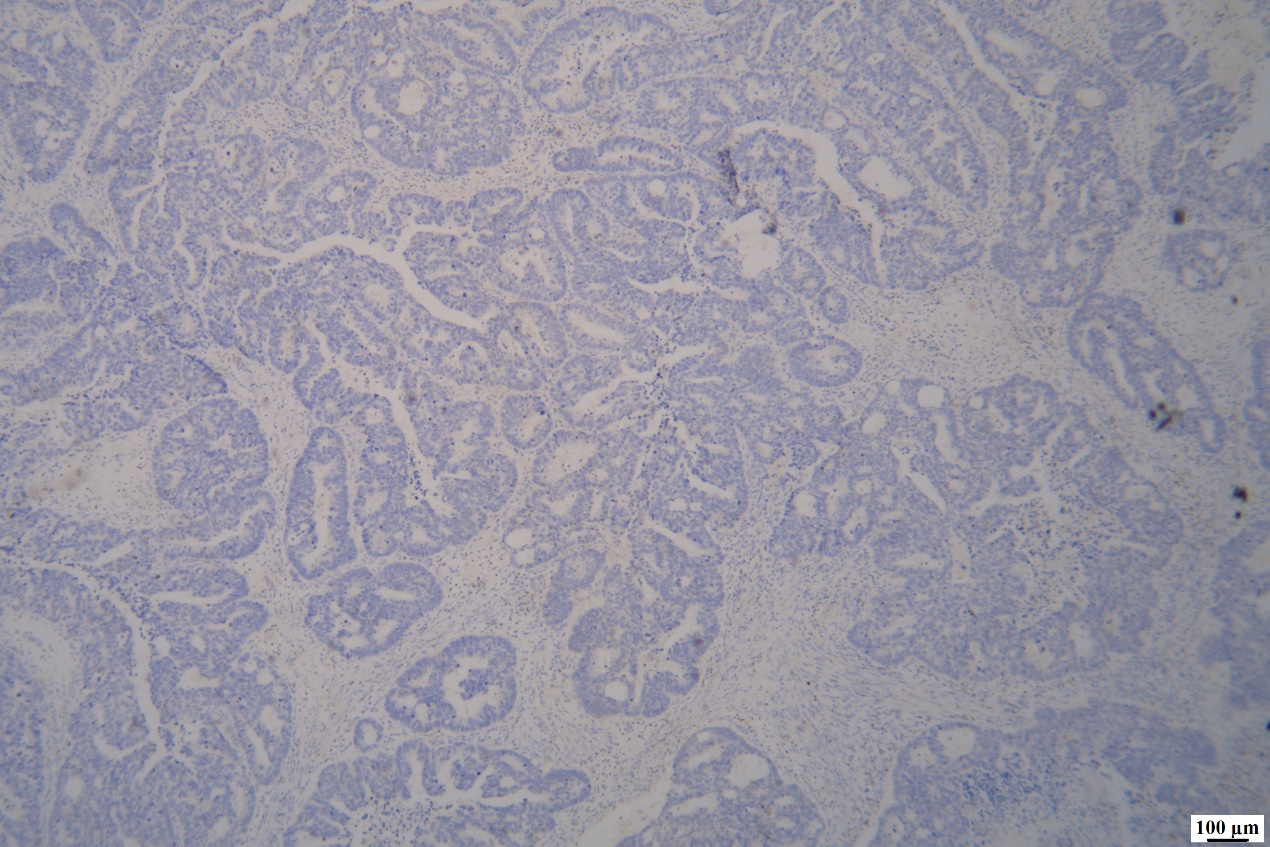

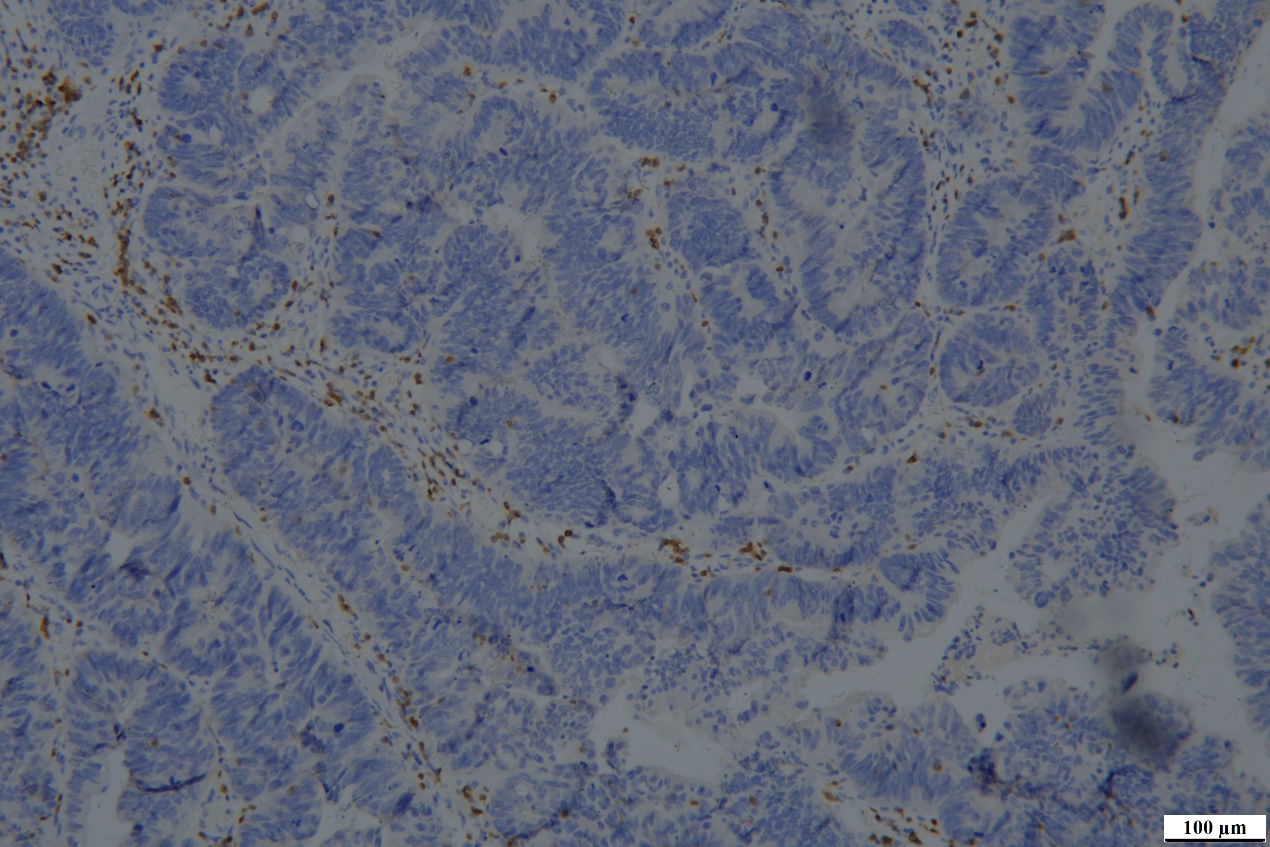

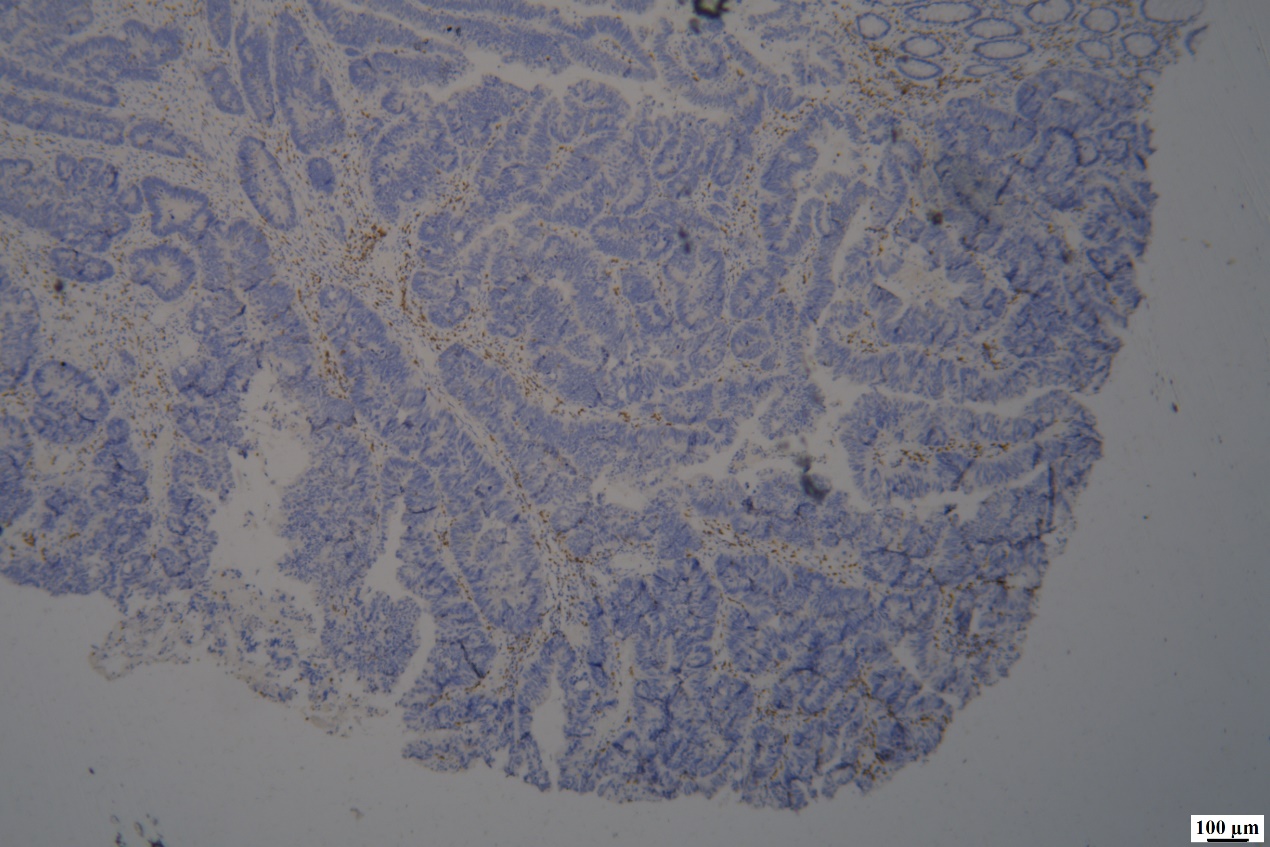

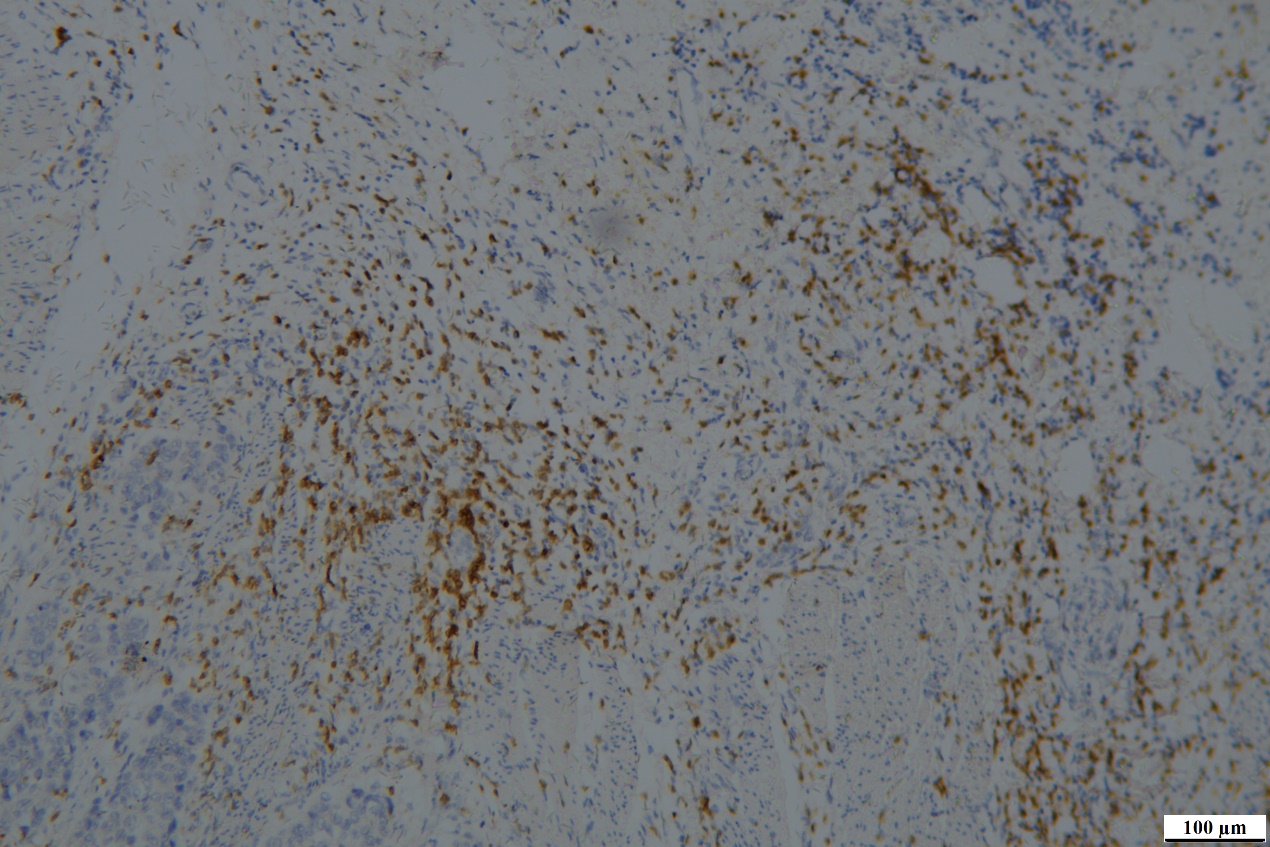

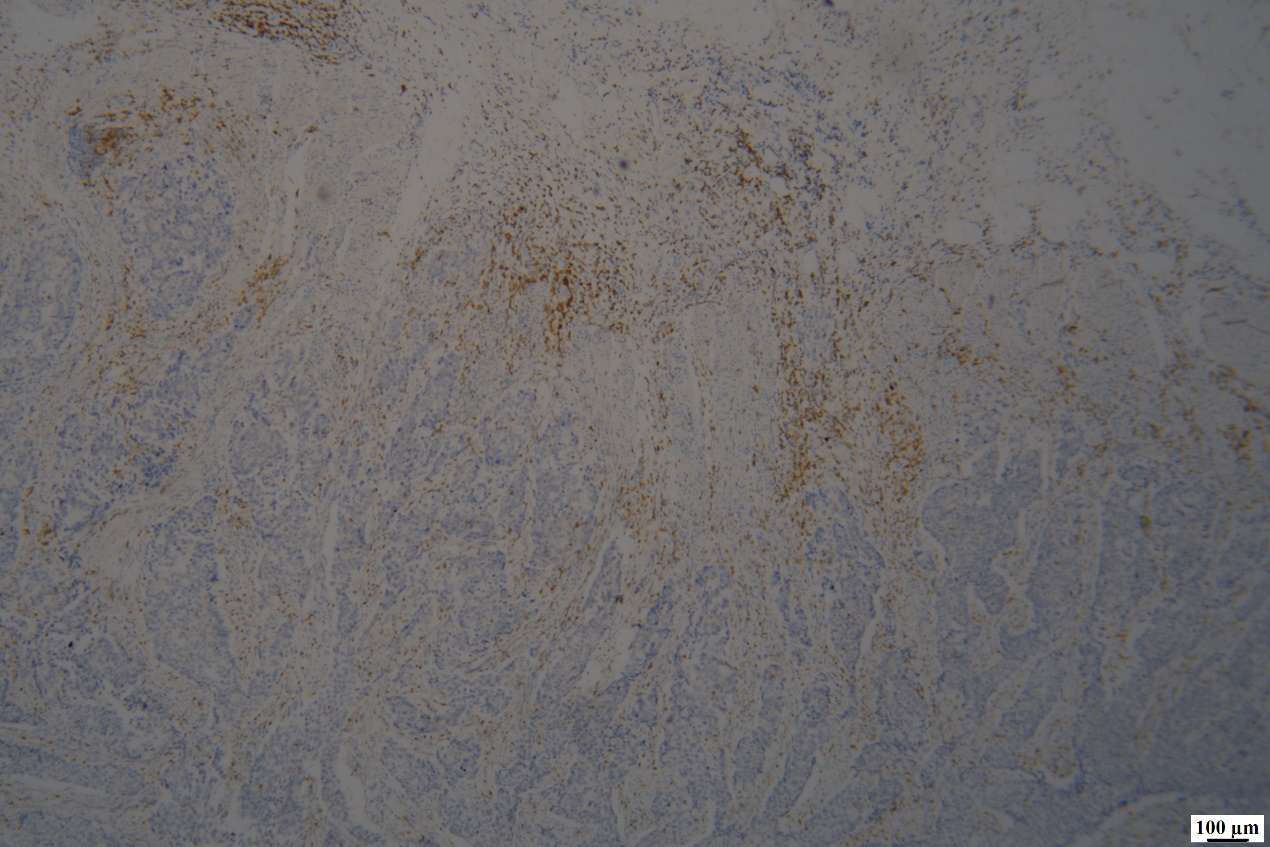

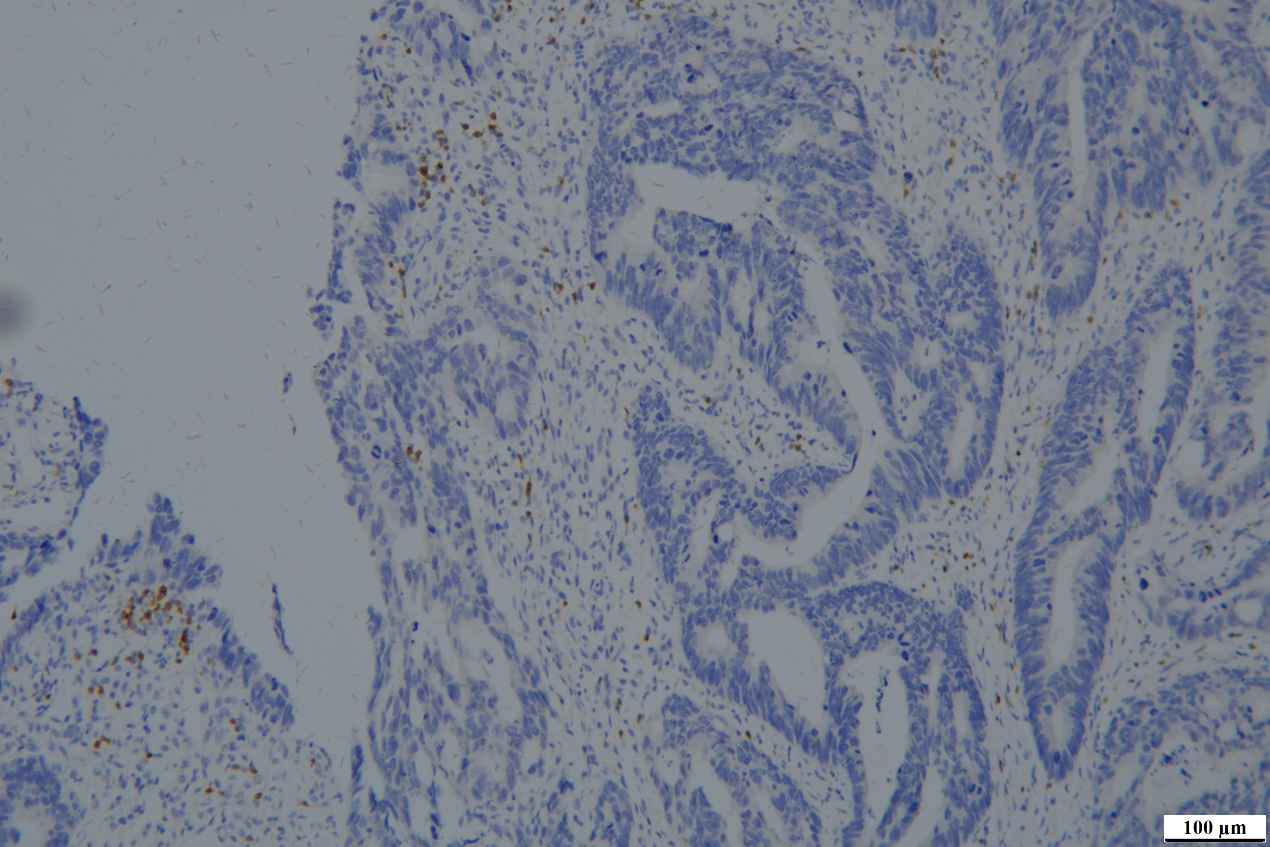

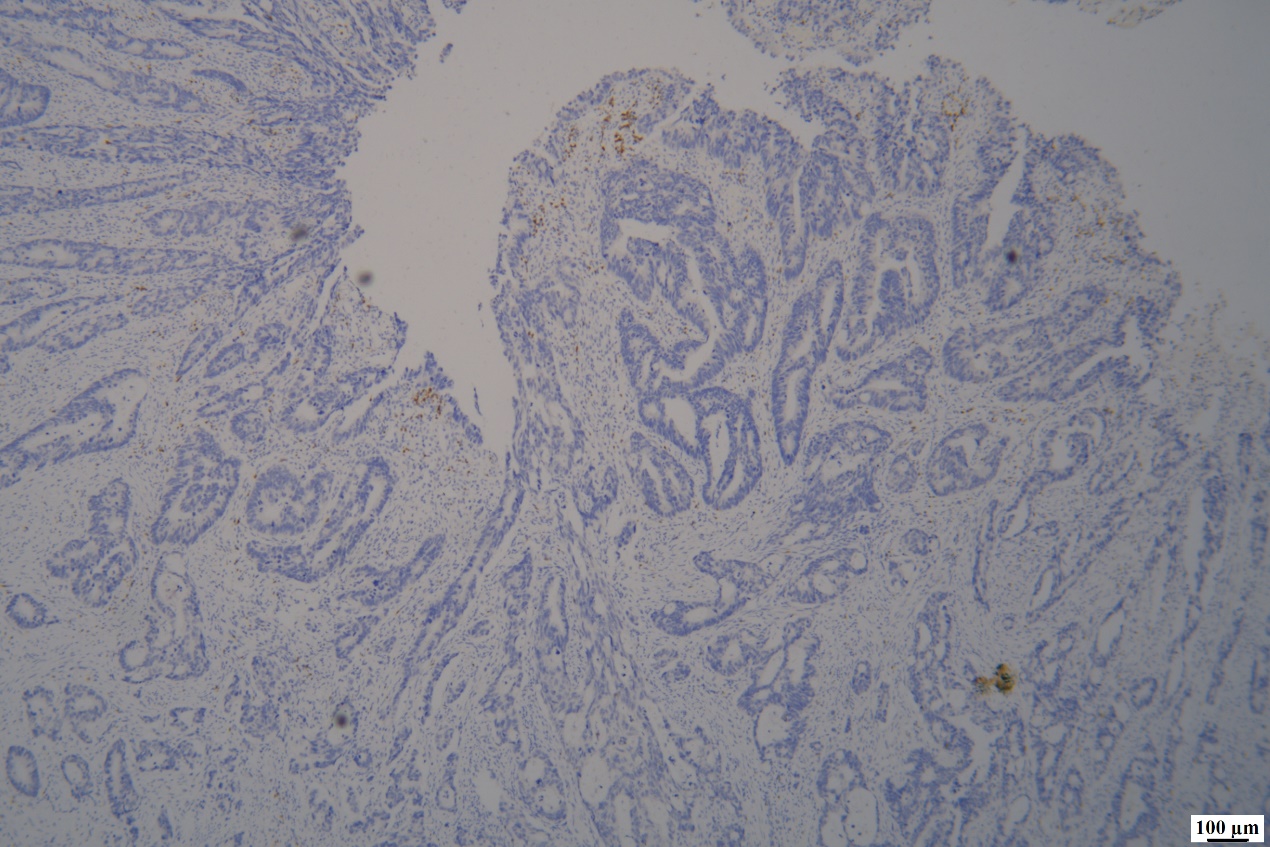

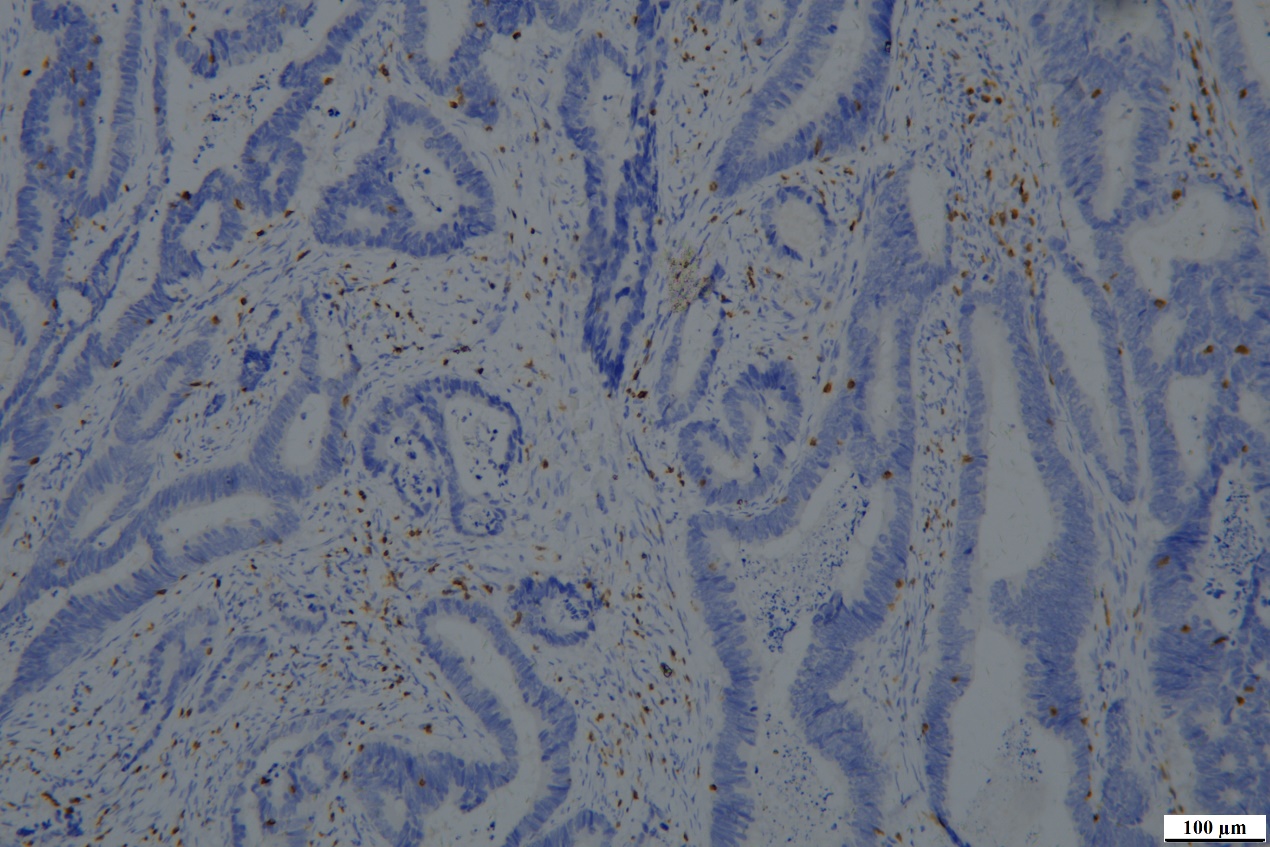

Supplement: Supplementary file 2 [file DataSheet2.docx]
